# Antifungal Activity of Essential Oils from Five *Piper* Species against *Botrytis cinerea* from Strawberry

**DOI:** 10.1021/acsomega.6c07389

**Published:** 2026-10-07

**Authors:** Inaiara Casapula, Diones Krinski, Juliana N. Maia, Henrique da S. S. Duarte, Francisco de A. Marques, Beatriz Helena L. N. Sales Maia

**Affiliations:** † Departamento de Química, 28122 Universidade Federal Do Paraná, Av. Cel. Francisco H. dos Santos, 100, Jardim Das Américas, Curitiba, Paraná 81530-900, Brazil; ‡ Curso de Ciências Biológicas, 67845 Universidade do Estado do Mato Grosso, Rodovia MT-358 Km 7, Jardim Aeroporto, Tangará da Serra, Mato Grosso 78300-000, Brazil; § Departamento de Fitotecnia e Fitossanidade, Universidade Federal do Paraná, Rua dos Funcionários, 1540, Cabral, Curitiba, Paraná 80035-050, Brazil

## Abstract

Strawberry (*Fragaria* × *ananassa* Duch.) is susceptible to several fungal diseases, such as gray mold, caused by *Botrytis cinerea*, that compromise fruits during and after harvest. Disease control usually involves synthetic fungicides; however, the frequent selection of isolates resistant to fungicides and the risk of chemical residues in fruits have motivated the search for more sustainable alternatives. Plant essential oils are promising due to their antimicrobial properties. This study analyzed the seasonal composition of the essential oils of five Brazilian *Piper* species (*Piper aduncum*, *Piper amalago*, *Piper caldense*, *Piper mikanianum*, and *Piper mollicomum*) and their effectiveness in controlling *Botrytis cinerea*. The chemical composition, determined by Gas Chromatography–Mass Spectrometry, revealed the main compounds as a function of seasonality, with myristicin and germacrone being the most prominent. The essential oils of *Piper caldense* and *Piper mikanianum* were the most effective in controlling *Botrytis cinerea*, with a minimum inhibitory concentration of 20 μL mL^–1^, surpassing that of the commercial fungicide mancozeb. These results suggest a sustainable alternative for fungal disease control in agriculture.

## Graphical abstract

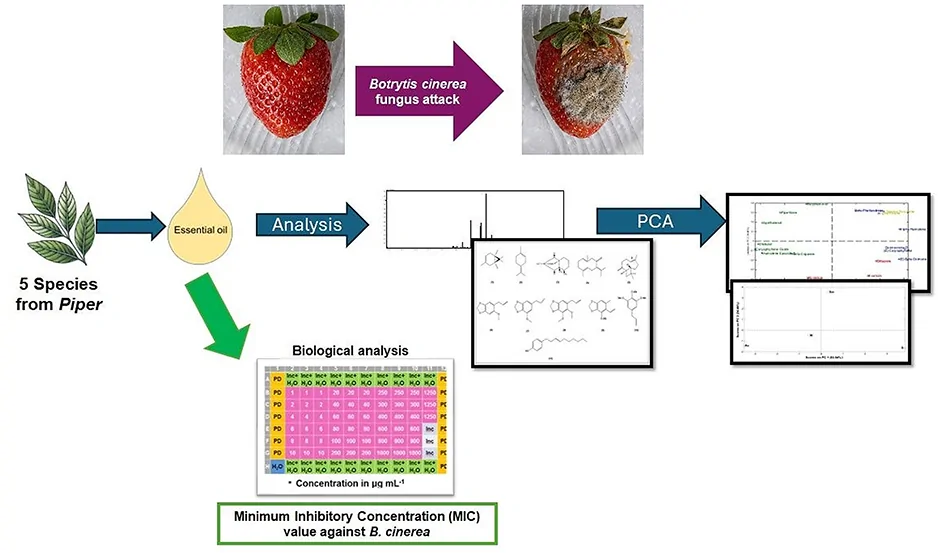

## 1 Introduction

The strawberry (*Fragaria* *×* *ananassa* Duch.) is a highly valued crop worldwide.^1–3^ Its cultivation is severely affected by fungal diseases, particularly gray mold (*Botrytis cinerea* Pers.:Fr). According to economic and scientific criteria, this species ranks second among the ten most important fungi worldwide because of its broad host range and its capacity to cause damage both during and after harvest.^4^ Unlike host-specific pathogens, *Botrytis cinerea* is a devastating necrotrophic fungus capable of infecting over 1,400 plant species, including high-value crops, vegetables, and ornamentals. Its impact is not restricted to fruit rot; the pathogen also damages leaves, flowers, seeds, and the plant crown both pre- and post-harvest.^5^ The disease affects several parts of the plant, including flowers,^5^ fruits, leaves, and the crown.^6^ In fruits, the typical symptoms include the appearance of brownish, non-watery lesions with poorly defined margins, followed by the development of a gray mass corresponding to the mycelium and conidia of the pathogen.^3,7,8^

The use of fungicides has been the main strategy to sustainable disease control, due to its genetic variability, adaptability, and abundant spore production, *B. cinerea* has a high risk of developing resistance.^9^

Inadequate management, including successive applications of fungicides with the same active ingredient or mode of action, leads to increased selective pressure for resistant populations of *B. cinerea*.^10^ The emergence of cases of resistance of *B. cinerea* to synthetic fungicides^11^ and the risk of residues in food have driven the search for sustainable disease control alternatives in plants.^12,13^

Alternative methods for disease control have been developed, focusing on less harmful treatments, aiming to reduce dependence on conventional synthetic fungicides.^14,15^ Among the various strategies, essential oils (EO) stand out. Essential oils are volatile secondary metabolites produced by plants, which protect against numerous pathogens and insects while aiding in coping with environmental stresses, such as excessive solar radiation.^16,17^ Plant-based EO are promising solutions that have been effective in protecting crops, particularly by controlling postharvest diseases^18^ through their fungitoxic action.

The genus *Piper* is one of the most relevant representatives of basal angiosperms, with an estimated 299 species, of which 184 are endemic.^19^ Species of the genus *Piper* are widely distributed in the tropical and subtropical hemispheres, contributing to their widespread popular use in cooking, aromatology, ornamentation, and medicine.^20,21^ *Piper* EO stands out for their significant activity against several phytopathogens. Studies have demonstrated the potential for inhibition of phytopathogens, as in the cases of *Piper aduncum* EO against *Colletotrichum musae* ^22^ and *Piper auritum* EO against *Fusarium moniliforme*, *Aspergillus ochraceus*,^23^ *Colletotrichum acutatum*, and *Botryodiplodia theobromae*.^24^

This study aims to analyze the seasonal composition of EO from five species endemic to Brazil: *Piper aduncum*, *Piper amalago*, *Piper caldense*, *Piper mikanianum*, and *Piper mollicomum*, and their potential to inhibit the phytopathogen *Botrytis cinerea* in strawberry. Unlike previous studies that focus solely on chemical characterization, this research integrates seasonal variability with bioactivity assays to identify the optimal harvest period, aiming for an efficacy that surpasses commercial synthetic standards.

## 2 Results and Discussion

Analysis of the *Piper* essential oils (EO) obtained by hydrodistillation from the leaves of the studied species revealed that *Piper mikanianum* EO exhibited high yields during summer (2.54 ± 0.32%) and autumn (2.21 ± 0.27%). In the case of *Piper aduncum*, the yields corroborate the values reported in previous studies, ranging from 0.96% to 1.43% according to Pino et al.,^25^ and from 1.20% to 3.40% according to Maia et al.,^26^ both referring to samples from the Amazon region. A lower yield occurred during autumn. *Piper amalago* also presented a positive yield, with considerable values in spring (1.38 ± 0.14%) and autumn (1.12 ± 0.07%). Figure 1 presents the EO yields of *Piper* species.

**Figure 1:**
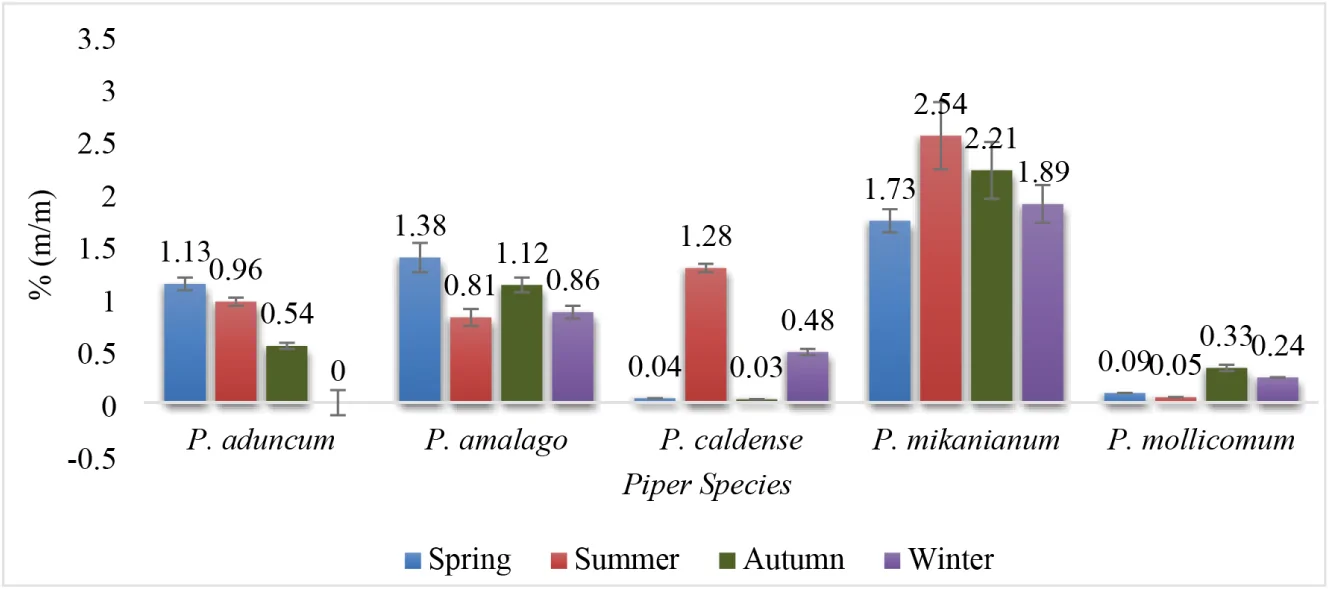
Seasonal variation in the EO yields (% m/m) of five *Piper* species. Values are expressed as the mean ± standard deviation of three independent replicates (n = 3).

The essential oil yield of *Piper caldense* peaked in summer (1.28 ± 0.04%), whereas yields in other seasons remained below 0.48% and were considered negligible. *Piper mollicomum*, in turn, presented the lowest yields in all seasons, with values between 0.05% and 0.33%. The highest performance occurred during autumn (0.33 ± 0.03%).

These data indicate that species such as *P. aduncum*, *P. mikanianum*, and *P. amalago* are promising for large-scale EO production, while others, such as *P. caldense* and *P. mollicomum*, have limitations due to low yields, except for some seasonal peaks.

The analysis of *Piper* EO obtained from the leaves of the studied species revealed that yield, and composition vary in a species-specific manner, without a uniform seasonal pattern across the genus. These fluctuations are driven by complex interactions between environmental factors—such as temperature, precipitation, and solar radiation—and plant genetics or phenology. For instance, summer conditions in Brazil (high temperatures and radiation) often enhance metabolic activity, explaining the yield peak of *P. caldense* (1.28%) during this season. Conversely, transition to colder or drier periods (autumn and winter) can induce physiological shifts toward heavier compounds like oxygenated sesquiterpenes. Similar qualitative stability of major compounds despite minor variations under seasonal fluctuations has been documented for *P. amalago* ^27^ and *P. aduncum* ^28^ demonstrating that seasonality quantitatively modulates metabolic profiles rather than altering the core chemical signature of the species

### 2.1 Chemical Composition

Table 1 summarizes the major constituents of the EO extracted from the leaves of the five *Piper* species investigated, as determined by GC–MS analysis, whereas the complete chemical composition, including all identified compounds, is provided in Table S1 of the Supporting Information. In the EO of *P. aduncum*, 30 compounds were identified, accounting for 86.92% of the total composition. Arylpropanoids represented the predominant chemical class, with myristicin, dillapiole, and asaricin identified as the major constituents (Figure 2). These compounds have also been reported in EO of *P. aduncum* collected in the Amazon region.^25,26^ Similarly, Potzernheim et al.^27^ reported variations in the EO composition among *P. aduncum* individuals collected from four locations in the Brazilian Federal District. This pattern is consistent with the chemical variability expected among native plants collected randomly and at different developmental stages.

**Table 1:** Major Constituents of the EO of Five *Piper* Species Collected During Different Seasons^a^

No	AI^1^	AI^2^	Identified compound	S	SM	A	W
*Piper aduncum*							
1	1498	1495	asaricin	30.23 ± 1.03	18.92 ± 1.48	24.95 ± 0.13	23.90 ± 0.53
2	1524	1517	myristicin	15.71 ± 2.10	10.68 ± 0.75	17.63 ± 0.30	16.68 ± 1.63
3	1623	1620	dillapiole	7.40 ± 0.97	2.87 ± 0.85	3.04 ± 0.08	7.92 ± 1.83
*Piper amalago*							
1	1009	1008	δ-3-carene	33.93 ± 8.48	9.32 ± 0.06	31.90 ± 4.35	26.01 ± 4.62
2	1463	1465	ishwarane	21.21 ± 1.24	26.65 ± 0.3	14.08 ± 0.98	19.9 ± 5.54
*Piper caldense*							
1	1524	1517	myristicin	32.46 ± 4.57	74.51 ± 11.89	16.17 ± 0.00	62.3 ± 6.77
*Piper mikanianum*							
1	1560	1555	(*E*)-elemicin	21.40 ± 5.28	3.10 ± 1.09	16.80 ± 0.33	7.73 ± 0.80
2	1656	1653	4,6-dimethoxy-5-vinyl-1,2-benzodioxole	17.43 ± 2.67	19.87 ± 2.41	13.07 ± 0.54	21.88 ± 1.06
3	1694	1693	germacrone	34.35 ± 5.28	50.73 ± 5.46	56.98 ± 1.13	44.74 ± 3.43
*Piper mollicomum*							
1	1009	1008	δ-3-carene	14.43 ± 4.28	-	-	1.89 ± 0.20
2	1611	1608	humulene epoxide II	0.11 ± 0.05	0.63 ± 0.09	25.47 ± 3.82	0.11 ± 0.09
3	1971	–	gibbilimbol B	7.10 ± 2.74	68.01 ± 7.46	-	9.96 ± 2.53

a AI^1^ - experimental arithmetic index; AI^2^ -literature arithmetic index. Values are presented as mean ± standard deviation (n = 3) of the relative peak area (%). S—spring; SM—summer; A—autumn; W—winter; and dashes (—) indicate that the compound was not detected in the sample.

**Figure 2:**
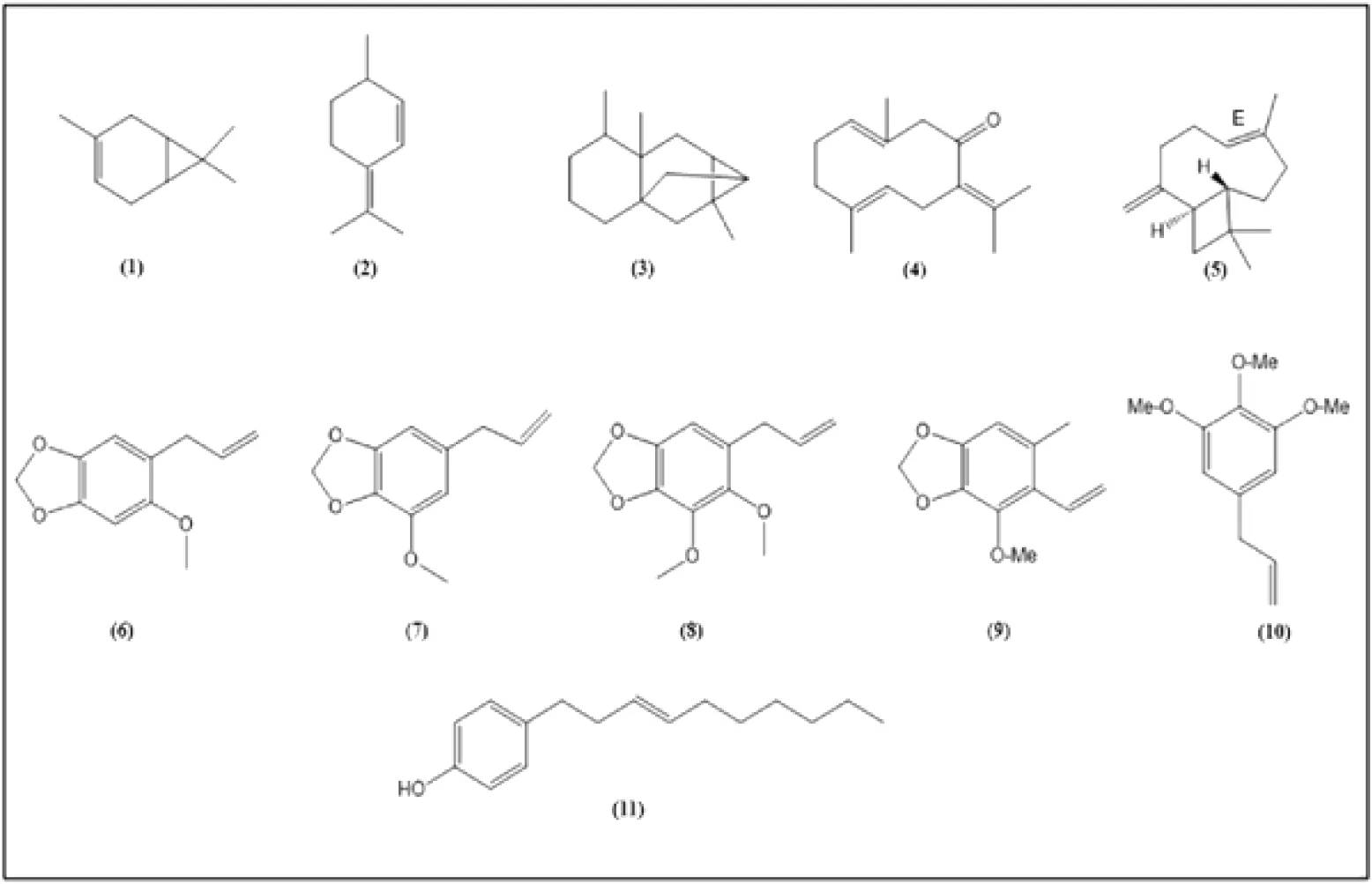
Major compounds of the EO from five *Piper* species. 1 – δ-3-carene; 2 – terpinolene; 3 – ishwarane; 4 – germacrone; 5 - (*E*)-caryophyllene; 6 – asaricin; 7 – myristicin; 8 – dillapiole; 9–4,6-dimethoxy-5-vinyl-1,2-benzodioxole; 10 – elemicin; and 11 – gibbilimbol B.

Almeida et al.^28^ suggested the possible occurrence of two chemotypes in *P. aduncum* based on variations in EO composition. In that study, arylpropanoids predominated in the Amazon Forest chemotype, whereas terpenes were the major constituents in the Atlantic Forest chemotype. This difference was attributed to abiotic factors. However, the occurrence of the same chemotype in four populations from the same location was attributed to genetic factors.^28^ These findings demonstrate that *P. aduncum*, even when growing in geographically close environments, may produce distinct secondary metabolites.

For *P. amalago*, 36 compounds were identified, representing 88.67% of the OE composition. The δ-3-carene, a monoterpene, and ishwarane, a sesquiterpene, were the major constituents (Figure 2). EO of *P. amalago* from populations collected in regions of São Paulo State, Brazil, showed variation in the relative abundance of major compounds, reaching 26% for *β*-phellandrene. Overall, the main constituents reported for this species are predominantly monoterpenes and sesquiterpenes, including *β*-phellandrene, *α-*pinene, germacrene D, sabinene, myrcene, bicyclogermacrene, *γ*-muurolene, and spathulenol.^3^

For *P. caldense*, 45 compounds were identified, corresponding to 90.20% of the EO composition. Myristicin was the major constituent in summer (Figure 2). Marked seasonal variation was observed in the EO profile, with higher proportions of this compound in winter (62.30%) and summer (74.51%). Previous reports indicate that *P. caldense* EO from Paraná State do not contain arylpropanoids, consisting exclusively of terpenes.^29^ Similarly, the EO of *P. caldense* from Pernambuco State contained 28 compounds, with *α*-cadinol (19.0%) as the predominant constituent, and likewise lacked arylpropanoids.^30^ These results suggest substantial geographic variation in the chemical profile of this species.

For *P. mikanianum*, 13 compounds were identified, representing 98.75% of the OE composition. Germacrone (34.35–56.98%), 4,6-dimethoxy-5-vinyl-1,2-benzodioxide (13.07–21.88%), and elemicin (3.10–21.40%) were the major constituents (Figure 2). Oxygenated sesquiterpenes predominate, followed by arylpropanoids. In contrast, EO of *P. mikanianum* previously reported for Atalanta, Santa Catarina State, showed high concentrations of safrole, reaching 80% of the total composition.^31^ This comparison reinforces the pronounced chemical variability of the species across different geographic origins.

A total of 40 compounds, accounting for 88.67% of the composition of *Piper mollicomum* EO, were identified. In this case, NMR spectroscopy was required to identify the major constituent (68%), since it was not found in the OE databases. These spectroscopic data are consistent with literature values reported for gibbilimbol B isolated and purified from extracts of *Piper malacophyllum* ^32^ and *Piper gibbilimbum*.^33^ This agreement confirmed the direct identification of gibbilimbol B as the major constituent in the crude essential oil of *P. mollicomum* without prior purification.

The following signals were observed in the ^1^H NMR spectrum (Supporting Information, Figure S16), the following signals were observed: δ 0.88 (t, *J* = 7.0 Hz, 3H, CH_3_); δ 1.27 (m, 8H, CH_2_); δ 1.96 (m, 2H, CH_2_); δ 2.26 (m, 2H, CH_2_); δ 2.59 (t, *J* = 7.0 Hz, 1H, CH); δ 5.47 (m, 3H, CH=CH); δ 6.74 (d, *J* = 8.6 Hz, 1H, Ar–H); and δ 7.04 (d, *J* = 8.6 Hz, 1H, Ar–H).

The ^13^C NMR spectrum (Supporting Information Figure S17) showed signals at δ14.10 (C10′), assigned to the terminal methyl carbon; δ 22.64 (C9′), 29.80 (C8′), 29.53 (C7′), 32.57 (C3′), and 34.69 (C6′), corresponding to methylene carbons along the alkyl chain; δ 31.75 (C2′) and 35.25 (C1′), assigned to carbons adjacent to the aromatic ring; δ 115.04 (C2, C6) and 129.32 (C3, C5), corresponding to aromatic carbons indicative of a *para*-substituted system; and δ 129.50 (C4′) and 131.14 (C5′), assigned to the olefinic carbons of the alkyl chain. These data agree with the values reported by Orjala et al. (1998),^33^ allowing the identification of gibbilimbol B as the major compound (Figure 2). The calculated arithmetic index (AI = 1971) has not yet been reported in the literature.

### 2.2 Seasonal Analysis of *Piper* EO

Principal component analyses (PCA) were performed using only component values 2% higher than the composition in the EO. This approach was adopted due to the high-value stratification and to facilitate graph interpretation. Even with the reduction, the variance values did not change.^34^ The scores and loadings graphs of the EO from *Piper* species are shown in Figures 3–7.

**Figure 3:**
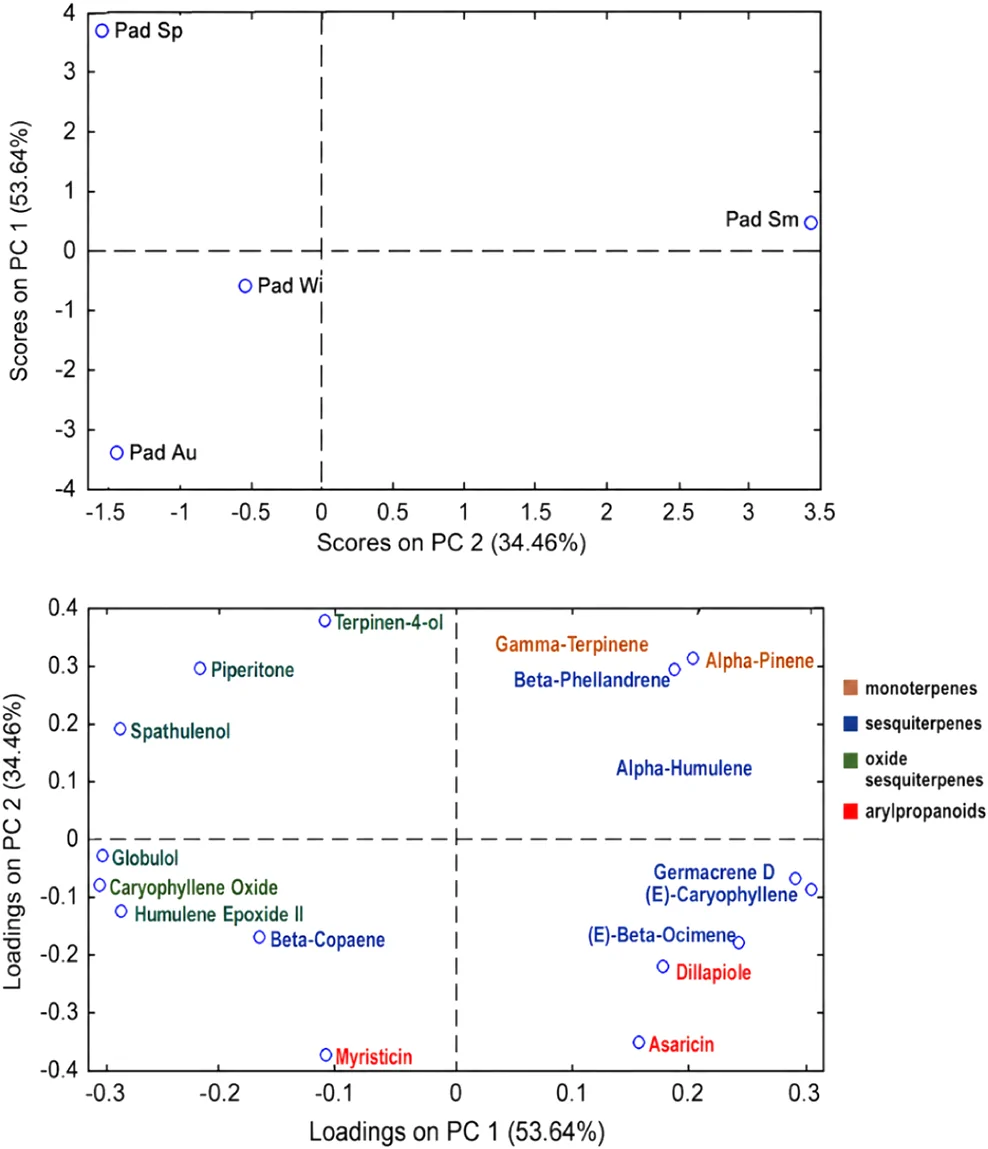
PCA scores and loadings plots for PC1 and PC2 of the EO from *P. aduncum* (Pad), showing seasonal variation in spring (Sp), summer, (SM), autumn (Au), and winter (Wi).

For *P. aduncum* EO, principal component 1 (PC1) explains 53.64% of the variance, while principal component 2 (PC2) explains 34.46%, totaling 88.10% of the variance between seasons (Figure 3). In spring, a predominance of arylpropanoids was observed. In winter, there is no influence of any variable; in autumn, oxygenated sesquiterpenes make their distinction, remaining in the negative quadrant of PC1 and PC2. In summer, monoterpenes have a significant weight in the variable. The opposite direction of the variables shows different properties, as observed in the autumn and spring analyses, something that occurs in the EO of *P. aduncum*.^35^

Remarkable differences between seasons were observed: in winter, there is a reduction in the production of arylpropanoids and, in spring, an increase in this concentration. In the winter and autumn, there is a decrease in the production of dilapiol, showing a variation in the production of its major components. Thus, spring was chosen for testing due to the higher concentration of arylpropanoids in this period.

For the *P. amalago* EO, PC1 explains 64.14% of the variance, while PC2 explains 22.89%, totaling 87.03% of the variation between seasons (Figure 4). A differentiation among seasons was observed based on their composition. The separation along PC1 distinguished between winter and summer, while PC2 highlighted distinctions between winter, autumn, and spring. There was no difference between autumn and spring, as they remained in the same quadrant. Likewise, there was no distinction between these seasons due to the concentration of monoterpenes, which were more pronounced during this period.

**Figure 4:**
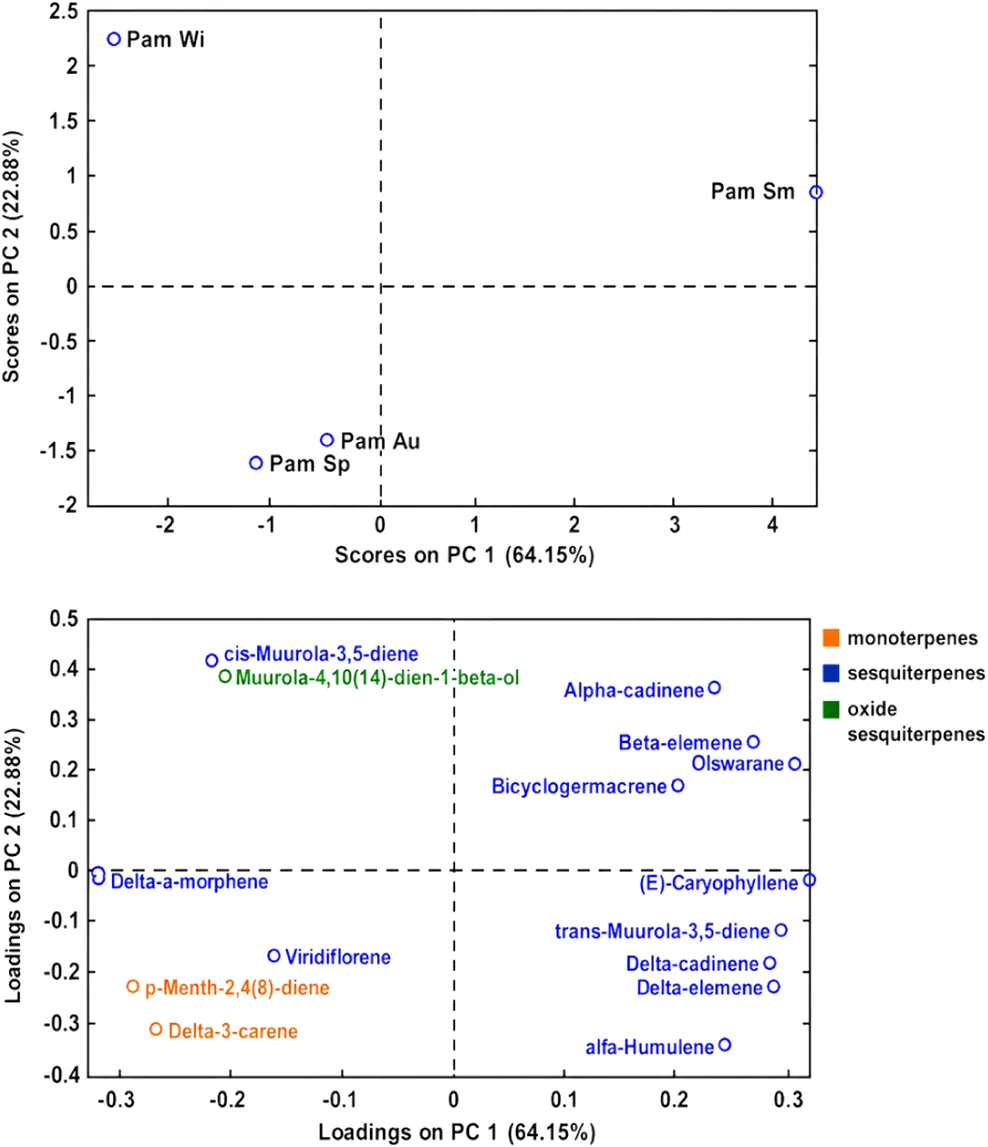
PCA scores and loadings plots for PC1 and PC2 of the EO from *P. amalago* (Pam), showing seasonal variation in spring (Sp), summer, (SM), autumn (Au), and winter (Wi).

For the *P. caldense* EO, PC1 accounts for 75.53% of the variance, while PC2 explains 14.02%, totaling 89.55% of the variation across the seasons (Figure 5). PC1 differentiates between autumn and summer, while PC2 highlights spring as the most distinct season. The compound that explains this differentiation is myristicin, which has a higher concentration in summer and winter, both appearing in the same negative quadrant of PC1 and remaining in the negative quadrant of PC2.

**Figure 5:**
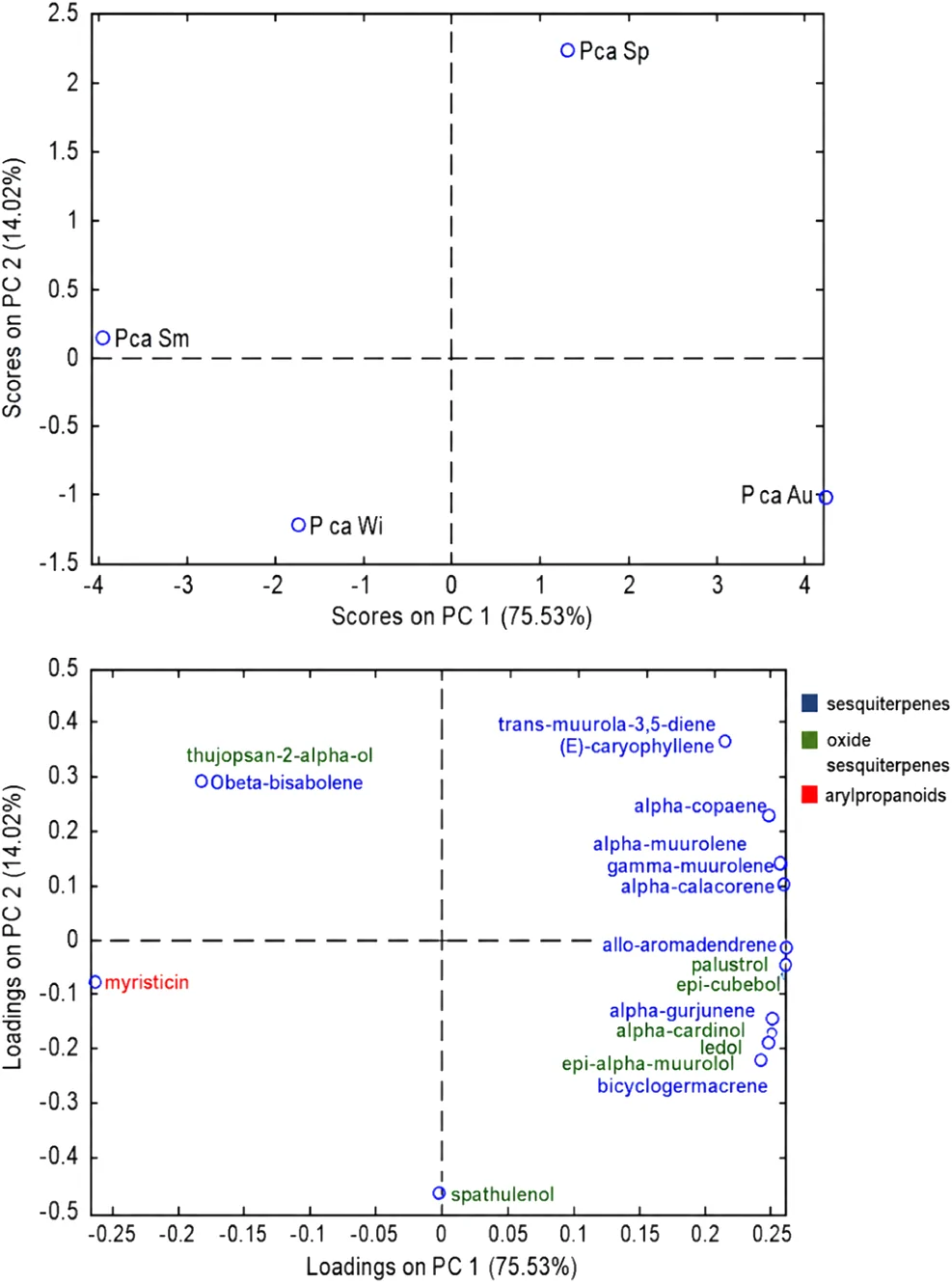
PCA scores and loadings plots for PC1 and PC2 of the EO from *P. caldense* (Pca), showing seasonal variation in spring (Sp), summer, (SM), autumn (Au), and winter (Wi).

For the *P. mikanianum* EO, PC1 explains 56.95% of the variance, while PC2 accounts for 32.42% of the variance, bringing the cumulative explained variance to 89.37% (Figure 6). Spring and summer are differentiated by PC1, and autumn stands out as the most distant season in the negative quadrant of PC2. The compounds responsible for this differentiation are bicyclogermacrene and valencene for spring, both located in the positive quadrant of PC1 and PC2.

**Figure 6:**
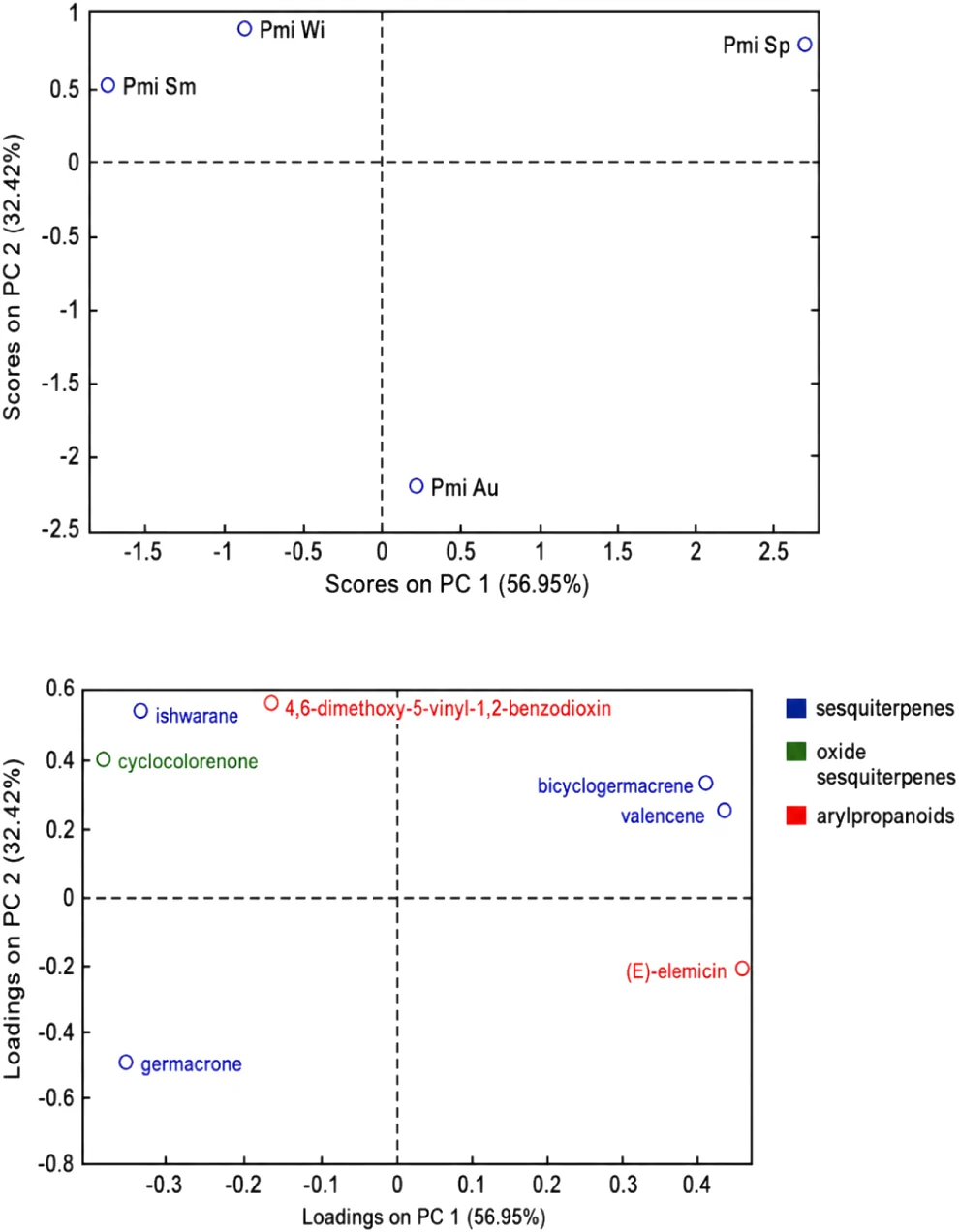
PCA scores and loadings plots for PC1 and PC2 of the EO from *P. mikanianum* (Pmi), showing seasonal variation in spring (Sp), summer, (SM), autumn (Au), and winter (Wi).

For the *P. mollicomum* EO, PC1 explains 50.31% of the variance, while PC2 accounts for 29.38%, totaling 79.69% of the variance between seasons (Figure 7). Summer is the only season standing out in the negative quadrant of PC2, attributed to the compound gibbilimbol B.

**Figure 7:**
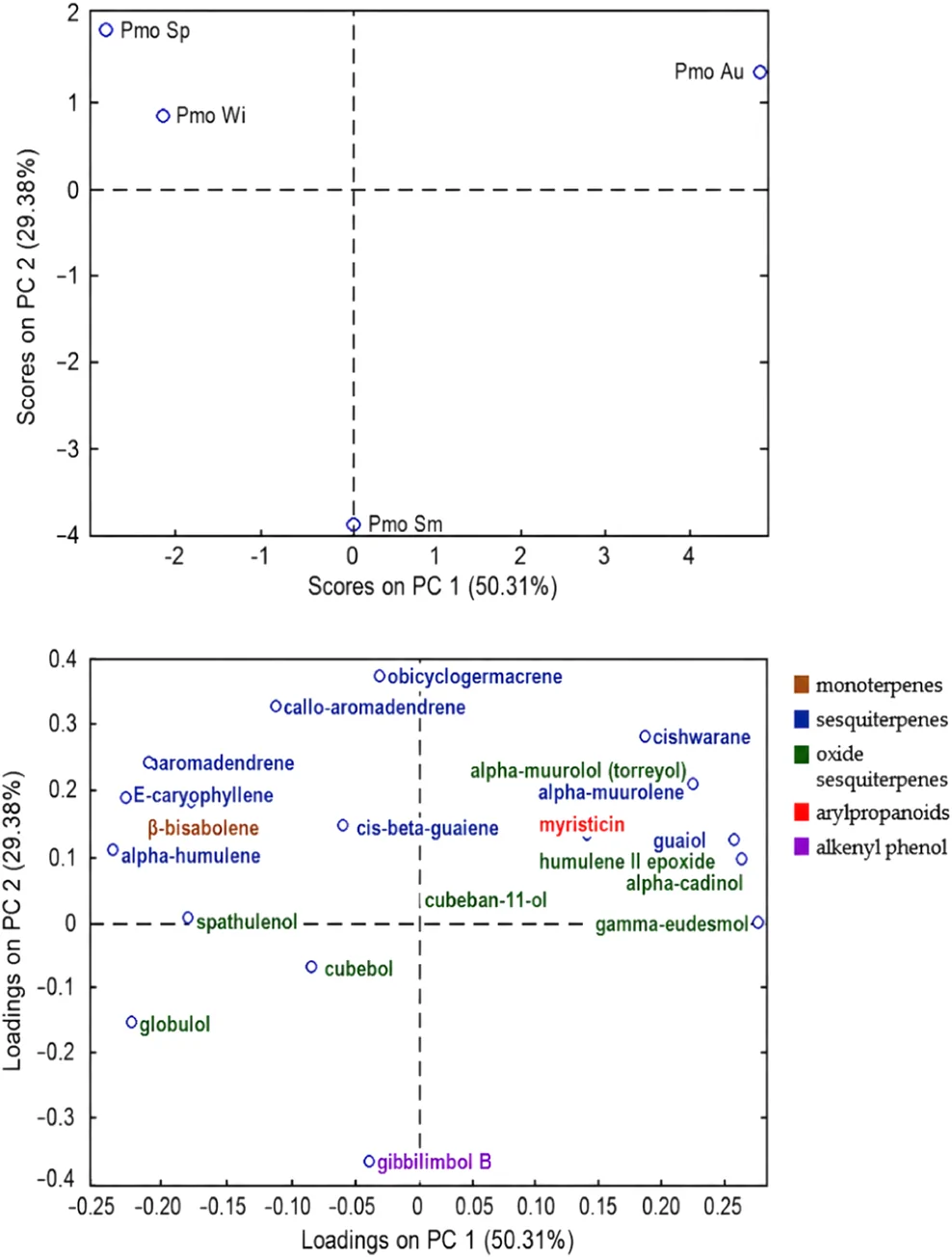
PCA scores and loadings plots for PC1 and PC2 of the EO *P. mollicomum* (Pmo), showing seasonal variation in spring (Sp), summer, (SM), autumn (Au), and winter (Wi).

Spring and autumn are differentiated by PC1, where a predominance of sesquiterpenes is observed in the negative quadrant of PC1, while oxygenated sesquiterpenes dominate near autumn in the positive quadrant of PC1.

Seasonal variation in EO composition results from the interaction among genetic, phenological, circadian, and environmental factors rather than from the influence of a single climatic variable. In *Piper* species, genotype and chemotype appear to determine the biosynthetic potential and the identity of the predominant constituents, whereas temperature, precipitation, solar radiation, and relative humidity mainly influence their relative abundance and EO yield. Assunção et al.,^35^ in a study of *P. aduncum* from the state of Rio de Janeiro, Brazil, showed that dillapiol remained the major compound during both the rainy and dry seasons and at different collection times. Although chemical richness and EO yield varied, no significant changes were observed in the relative abundance of the major constituent.^35^ Similarly, in *P. amalago* from the state of Espírito Santo, Brazil, *β*-elemene, germacrene A, linalool, and *β*-caryophyllene remained among the major constituents, although their relative proportions varied between the dry and rainy periods. These findings demonstrate that seasonality may quantitatively alter the chemical profile without necessarily changing its predominant chemical signature.^36^

In addition to abiotic environmental factors, phenology and endogenous temporal regulation may also influence specialized metabolism. These findings are consistent with the understanding that secondary metabolic pathways, including the arylpropanoid pathway, are regulated by environmental and climatic conditions and respond to drought periods, temperature, UV radiation, nutrient availability, and oxidative stress.^37^

Based on the EO composition results and seasonal evaluation, certain EO were selected for antifungal activity testing against *B. cinerea*. The selection of EO was based on the highest concentration of the major compounds, with spring being chosen for *P. aduncum* and *P. amalago*, summer for *P. caldense* and *P. mollicomum*, *and autumn for* *P. mikanianum*.

### 2.3 Antifungal Activity against *B. cinerea*

The MIC values observed for all treatments were identical across replicates within each experiment and between independent experiments. The MIC values for the *Piper* species showed a difference in the inhibitory activity of the EO against *B. cinerea*. Both *P. caldense* and *P. mikanianum* exhibited the strongest antifungal activity, with MIC values of 20 μL mL^–1^. These values were half that observed for the positive control, mancozeb with MIC value of 40 μL mL^–1^, clearly indicating that, under the *in vitro* conditions used in this study, the EOs of *P. caldense* and *P. mikanianum* were able to inhibit fungal growth at half the concentration required for mancozeb (Figure 8). These results demonstrate the potential of these EO for the *in vitro* control of the fungus studied. However, further studies are needed to validate their control efficacy and practical applicability under field and postharvest conditions. It is relevant to highlight that mancozeb and other fungicides are associated with high environmental impact and pathogen resistance.^38^ Our results position these EO as highly competitive candidates for bio-fungicide development. The MIC values obtained for all treatments in the antifungal assay are shown in Figure 9.

**Figure 8:**
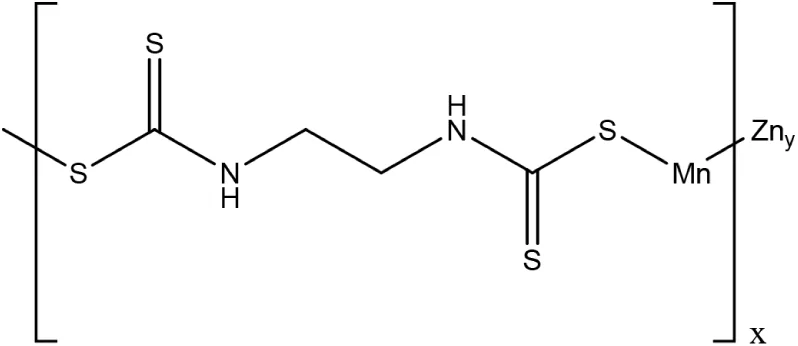
Mancozebe chemical structure.

**Figure 9:**
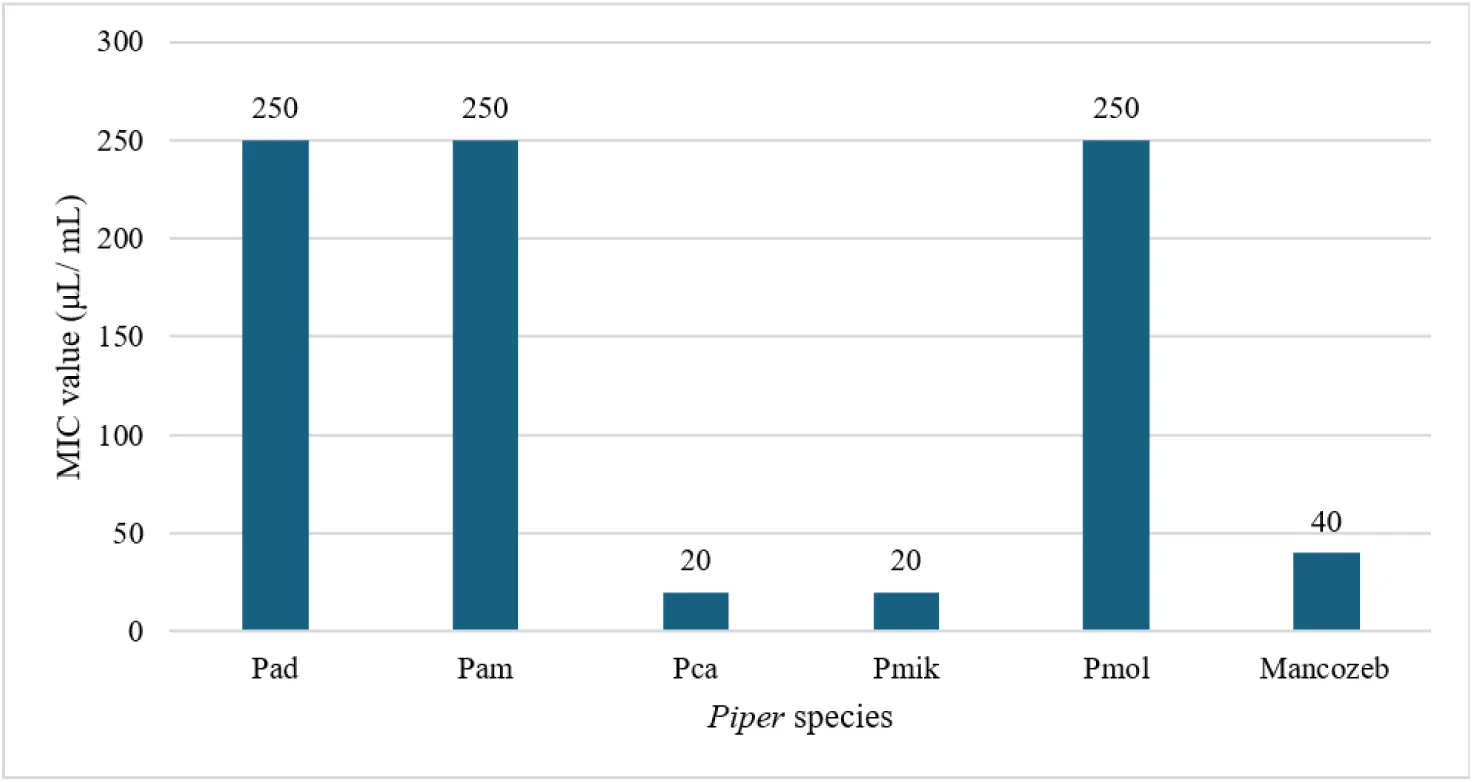
Inhibitory effects of EO from the leaves of *P. aduncum* (Pad), *P. amalago* (Pam), *P. caldense* (Pca), *P. mikanianum* (Pmik), and *P. mollicomum* (Pmol), mancozeb and negative control (NC) against *B. cinerea*.

Similar results have been reported for EO from other plant species against *B. cinerea*. Oliveira Filho et al.^39^ evaluated the EO of *Mentha piperita*, *Cymbopogon martinii*, *Cinnamomum camphora*, and *Mentha spicata* and reported MIC values of 10, 5, 20, and 5 μg mL^–1^, respectively. The authors also observed that selected EO were effective in reducing the incidence and severity of gray mold in inoculated strawberries, supporting their potential application for the control of *B. cinerea*. These findings^39^ are consistent with the antifungal potential observed in the present study for *P. caldense* and *P. mikanianum*, which exhibited the lowest MIC values among the *Piper* species evaluated.

For the negative control used to verify fungus behavior, an increase in optical density (OD) was observed in all tests after 96 h, indicating fungal growth. The MIC inhibition values appear along with the corresponding ODs observed at the EO concentrations, as described in Figure 10.

**Figure 10:**
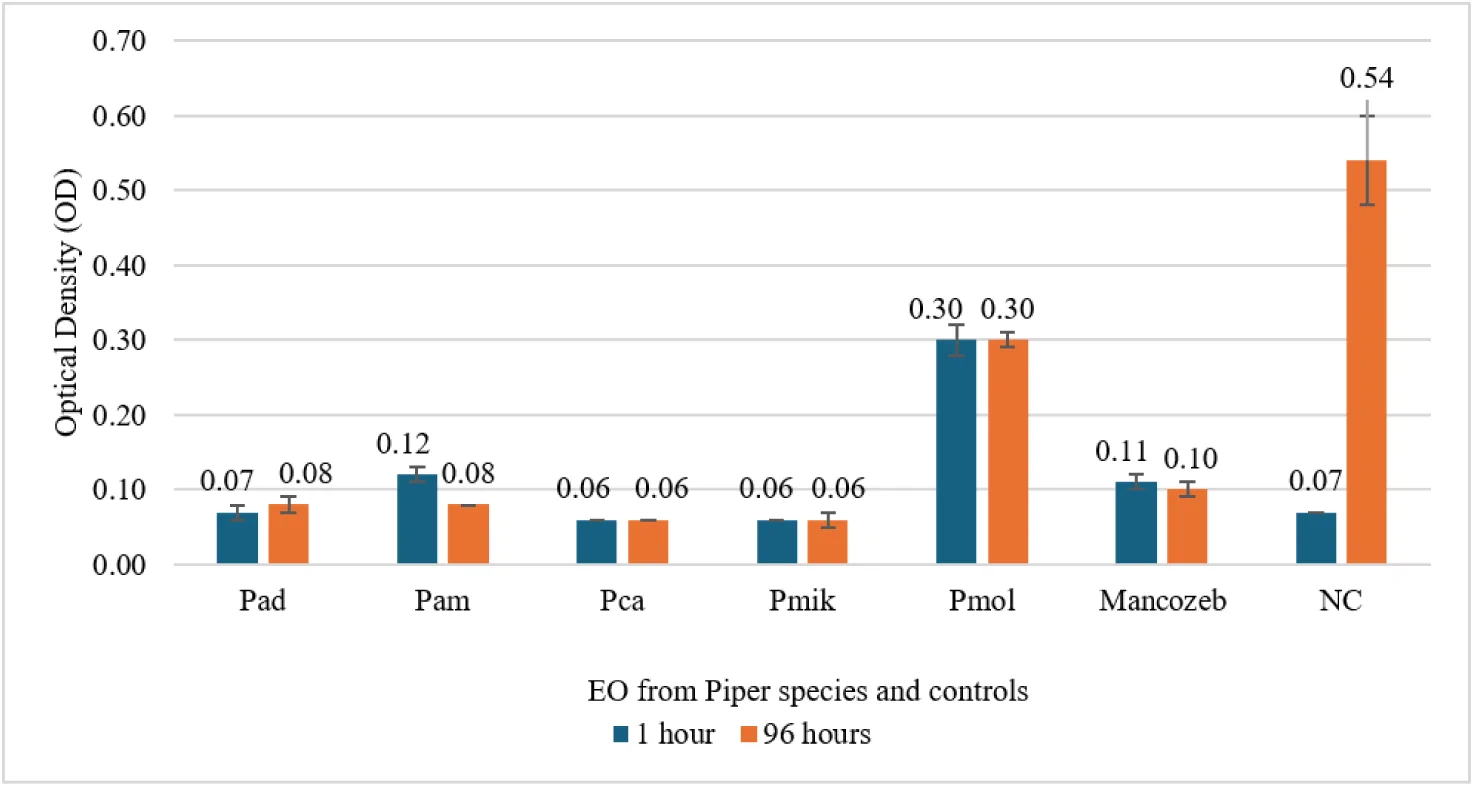
Optical density (OD) measured after 1 and 96 h of incubation in the antifungal assay against *B. cinerea* for the EO of *P. aduncum* (Pad), *P. amalago* (Pam), *P. caldense* (Pca), *P. mikanianum* (Pmik), and *P. mollicomum* (Pmol). Mancozeb and water/YPD medium were used as the reference fungicide (positive control) and negative control (NC), respectively. Values represent the mean ± standard deviation of three independent replicates (n = 3).

The EO may act on fungi through the hyphae. Consequently, EO cause rigidity and integrity loss of the hyphal cell wall and cytoplasmic components, which results in cell death.^40^ However, other antimicrobial mechanisms for EO components have been proposed, such as the disruption of enzymatic systems, lipids, proteins, and genetic material of the microorganism, as well as the coagulation of cytoplasm, electron flow, and active transport.^41^

The EO that yielded the best results contain arylpropanoids as major compounds, which have a double bond in the propenyl unit attached to the aromatic ring. Arylpropanoids have several reported antimicrobial activities,^42,43^ either when isolated or in combination.^44,45^ Carrasco et al.^46^ used 21 natural and synthetic arylpropanoids, with structures like safrole and eugenol, to evaluate their antifungal properties against a variety of human opportunistic pathogenic fungi. When comparing compounds with similar structures, it is possible to identify that the presence of the allyl group is crucial for the antifungal characteristic of these arylpropanoids.^45^

Although the antifungal activities reported in the literature support a possible contribution of the major arylpropanoids to the inhibitory effects observed in this study,^46–50^ the activity of a complex EO cannot be directly attributed to its most abundant constituents. Studies on arylpropanoids have shown that their antifungal activity is strongly influenced by molecular structure and by the substitution pattern of the aromatic ring and side chain.^46^ In particular, isolated dillapiole has exhibited antifungal activity against different phytopathogenic fungi, including *Botrytis cinerea*,^47^ whereas isolated myristicin has shown moderate antifungal activity against *Candida albicans*, *Candida tropicalis*, and *Pichia kudriavzevii*.^49^ Asaricin has also been reported in the literature as a arylpropanoid with antifungal activity.^50^ These findings support the hypothesis that the major arylpropanoids may contribute to the antifungal activity observed for the *Piper* EO. However, interactions among major and minor constituents may result in additive, synergistic, or antagonistic effects. Therefore, further studies using the isolated major compounds, both individually and in combination, under the same experimental conditions employed against *B. cinerea*, are required to determine their actual contribution to the antifungal activity of the *Piper* EO.

Regarding the potential organoleptic impact of postharvest treatment, the highly volatile nature of *Piper* EO suggests they would evaporate rapidly from the fruit surface, leaving no persistent flavor residues on the strawberries. Furthermore, the future incorporation of these EO into biodegradable edible coatings (such as chitosan) or nanoemulsions could allow a controlled release of volatile components, protecting the fruit while mitigating any sensory changes. From a toxicity perspective, the major active compounds identified (e.g., myristicin and germacrone) possess low toxicity compared to synthetic fungicides like mancozeb, which leave persistent, hazardous chemical residues

## 3 Conclusions

This study demonstrates that the essential oils of *Piper caldense* and *Piper mikanianum* possess strong in vitro antifungal activity against *Botrytis cinerea* in strawberries, outperforming the synthetic fungicide mancozeb. By linking seasonal yield dynamics with PCA chemical profiling, we identified myristicin and germacrone-rich profiles as key drivers of this bioactivity. Although these laboratory findings represent an initial step and cannot be directly extrapolated to field conditions, they highlight the potential of Brazilian *Piper* species as promising, sustainable sources for bio-fungicide development. Additionally, further cytotoxic and genotoxic investigations are required to establish the safety profile of these essential oils for agricultural and consumer use.

## 4 Materials and Methods

### 4.1 Plant Material

Voucher specimens were deposited in the Tangará Herbarium (TANG), at the Professor Eugênio Carlos Stieler University Campus of the University of the State of Mato Grosso, in the municipality of Tangará da Serra. Table 2 provides the collected species, collection dates, deposit numbers, and geographical coordinates.

**Table 2:** *Piper* Species, Dates, and Their Collection Location

	Date of Collections						
Species	Spring	Summer	Autumn	Winter	Voucher number	State/UF	Geographic coordinates
*P. aduncum*	Oct/15	Jan/16	Apr/15	Jul/15	6842	Novo Progresso/PA	14°37’29.0“S 57°29’09.1”W
*P. amalago*	Nov/15	Feb/16	May/15	Aug/15	6833	João Pessoa/PB	22°51’37.19“S 48°26’14.32”W
*P. caldense*	Sep/15	Feb/15	Apr/15	Jul/15	6838	Tapiraí/ SP	22°50’42.03“S 48°25’34.04”W
*P. mikanianum*	Sep/15	Feb/15	Apr/15	Jul/15	6866	São José dos Pinhais/PR	22°53’32.57“S 48°28’57.45”W
*P. mollicomum*	Sep/15	Feb/15	Apr/15	Jul/15	6863	Tapiraí/ SP	7°06’59.0“S 55°24’22.1”W

### 4.2 Essential Oil Extraction

The leaves were cleaned and dried in a circulating air oven at 40 °C until the material’s mass became constant, then coarsely ground. The dried leaves (50 g) were subjected to hydrodistillation in a modified Clevenger apparatus for 4 h, in triplicate. The yield of the EO is expressed as a percentage (m/m) using the following equation:

$$\%\;\mathrm{oil}\;\mathrm{yield}=\frac{\mathrm{mass}\;\mathrm{obtained}\;\mathrm{EO}\;\left(g\right)}{\mathrm{mass}\;\mathrm{of}\;\mathrm{plant}\;\mathrm{material}\;(g)}\times 100$$

### 4.3 Gas Chromatography-Mass Spectrometry (GC-MS) Analysis and Identification of Volatile Components

The composition of the volatile constituents of five *Piper* EO was analyzed by GC-MS using a Shimadzu TQ8040 GC-MS chromatograph (Kyoto, Japan) and an HP-5MS capillary column (30 m × 0.25 mm × 0.25 μm). A 1 μL aliquot of the 100 ppm EO solution in hexane was injected with a split ratio of 1:90, using helium as the carrier gas at a constant flow rate of 1.02 mL min^–1^. The initial oven temperature was set to 60 °C, increasing to 240 °C at a heating rate of 3 °C min^–1^. The injector temperature was set to 250 °C, and the interface temperature to 300 °C. The ionization energy and mass scan range were set to 70 eV and 40–400 amu, respectively. The retention indexes were calculated using a homologous series of *n*-alkanes with 8 to 30 carbon atoms, which were injected under the same conditions used for the OE. Compounds were identified by comparing their retention indices and mass spectra with those from the literature^51^ and the NIST 14.0 mass spectrometry library with a minimum compatibility of 90%. The quantification of the compounds in the EO was performed by GC-FID on an Agilent 7890A chromatograph, under the same conditions described for the GC-MS analysis.

### 4.4 Nuclear Magnetic Resonance (NMR) Spectroscopy

The ^1^H and ^13^C NMR spectra of the *Piper mollicomum* essential oil were recorded on an NMR spectrometer (Bruker) operating at 200 and 50 MHz, respectively. The analysis was performed directly on the crude essential oil without any prior chromatographic purification. The sample was dissolved in deuterated chloroform CDCl_3_, which also served as the internal lock. Chemical shifts (δ) are reported in parts per million (ppm) and coupling constants (*J*) are expressed in Hertz (Hz).

### 4.5 In Vitro Antifungal Activities of *Piper* Species

#### 4.5.1 Phytopathogen Isolation

*Botrytis cinerea* was isolated from the rotting fruit of strawberries provided by farmers from the city of São José dos Pinhais, state of Paraná, Brazil. The fungus was identified via molecular methods. Isolate DNA was extracted using the CTAB protocol, with some modifications. The species was identified using PCR with G3PDH-F2 forward primers and G3PDH-R reverse primers to identify *B. cinerea*.^52^ The isolate was deposited at the Laboratory of Epidemiology for Integrated Disease Management (LEMID) at the Federal University of Paraná.

#### 4.5.2 Production and Standardization of Fungal Inoculum

For fungal growth and sporulation, the isolate was subcultured onto Petri dishes containing potato dextrose agar (PDA) and incubated in a growth chamber at 25 ± 2 °C. After 7 days, 10 mL of sterile distilled water containing 0.1% Tween-80 (v/v) was added to the plates to obtain the spores. Conidium suspensions were adjusted with sterile saline to a final concentration of 2 × 10^5^ spores mL^–1^ using a Neubauer chamber and a light microscope (Zeiss AxioLab A1).

#### 4.5.3 Determination of Minimum Inhibitory Concentration (MIC)

MIC values for the *Piper* EO were determined using a quantitative assay in 96-well microtiter plates.^53^ For each *Piper* EO, three independent experiments were performed, each consisting of one plate with triplicate wells for each concentration. Figure 11 shows the concentrations of the EO from *Piper* species and their arrangement on the microplate. In each well, the EO solution was prepared using 5% dimethyl sulfoxide (DMSO) and 0.1% Tween 80, with concentrations ranging from 1 to 1200 μL mL^–1^. Autoclaved distilled water was then added to complete a final volume of 40 μL per well. Next, 160 μL of a *Botrytis cinerea* conidial suspension (2.0–2.5 × 10^5^ conidia mL^–1^) prepared in potato dextrose broth (PDB) was added to each well. The plates were incubated at 25 ± 2 °C under a 12 h photoperiod. Fungal growth was assessed by measuring absorbance at 650 nm during the first hour and again after 96 h using an automatic microplate reader (SpectraMax M2), the test was performed in triplicate.

**Figure 11:**
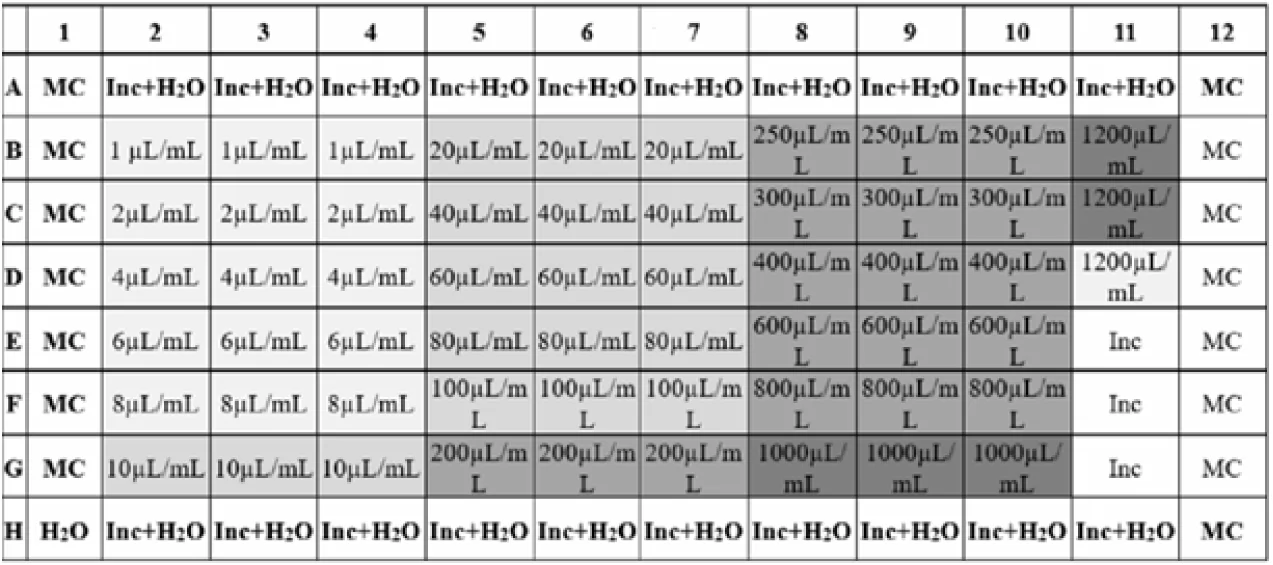
Diagram of the concentration distributions of *Piper* EO in the microdilution plates. *MC: PDB culture medium; INC +H_2_O: spore solution with a concentration of 2 – 2.5 × 10^5^ spores in PDB culture medium with the addition of water; INC: spore solution.

A comparison of the fungal inhibition results by the EO of the five species studied was conducted using the optical density measured in the first hour and at the end of the experiment. The comparison between these values allowed for the evaluation of fungal growth, in which a value equal to or lower than the initial one indicated inhibition of *B. cinerea* growth, enabling MIC determination. The fungicide mancozeb and 0.1% (v/v) Tween 80 were used as positive and negative controls, respectively.

### 4.6 Data Analysis

For the analysis of variance related to seasonality, Principal Component Analysis (PCA) was used. Multivariate data processing was performed using Excel 365, ORIGIN 8, and PLS-Toolbox 3.0 (Eigenvector Research, Inc.), operating in the Matlab 7.0 environment. All experiments were performed in triplicate (n = 3), and data were expressed as mean ± standard error, using descriptive statistics.

## Supplementary Material



## Notes

Insert DOI after publication: selected GC–MS chromatograms of EO from five *Piper* species collected in different seasons (Figures S1–S5); mass spectral comparisons between reference data reported by Adams^51^ and experimental GC–MS spectra of selected constituents identified in the EO (Figures S6–S15); ^1^H and ^13^C NMR spectra used for the structural characterization of gibbilimbol B (Figures S16–S17); a table identifying the constituents of EO from *Piper* species across different seasons (Table S1); and a table showing the inhibition of *B. cinerea* growth by EO obtained from *Piper* leaves (Table S2) (PDF)
