# Supplementary material for: Antifungal Activity of Essential Oils from Five Piper Species against Botrytis cinerea from Strawberry
Source: ACS Omega. 2026 Oct 7;11(41):63466–76. doi: 10.1021/acsomega.6c07389 (PMC13689280; doi:10.1021/acsomega.6c07389)
Supplement: Supplementary file 1 [file ao6c07389_si_001.pdf]

Supporting Information

Antifungal Activity of Essential Oils from five *Piper* species against *Botrytis cinerea* from strawberry

Inaiara Casapula[1], Diones Krinski[2], Juliana Nicolau Maia[3], Henrique da Silva Silveira Duarte[3], Francisco de Assis Marques[1] and Beatriz Helena Lameiro de Noronha Sales Maia[1*].

1 Departamento de Química, Universidade Federal do Paraná, Av. Cel. Francisco H. dos Santos, 100, Jardim das Américas, 81530-900 Curitiba, PR, Brazil.

2 Curso de Ciências Biológicas, Universidade do Estado do Mato Grosso, Rodovia MT-358 km 7, Jardim Aeroporto, 78300-000, Tangará da Serra, MT, Brazil.

3 Departamento de Fitotecnia e Fitossanidade, Universidade Federal do Paraná, Rua dos Funcionários, 1540, Cabral, 80035-050, Curitiba, Paraná, Brazil.

***Corresponding author:** E-mail: **noronha@ufpr.br**

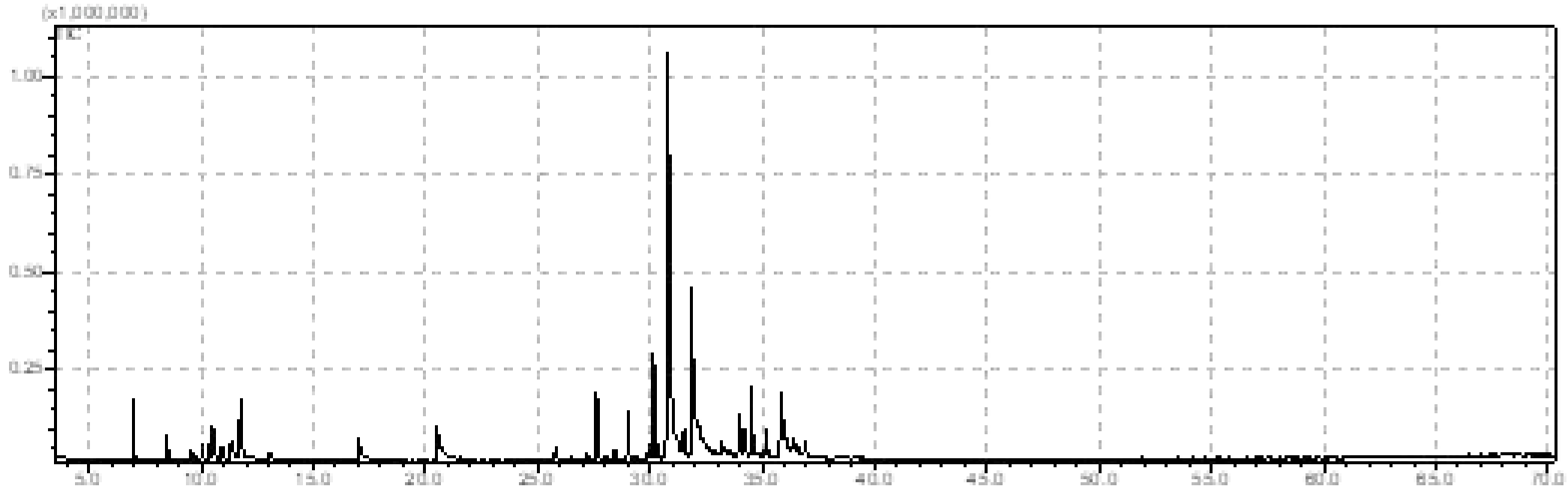

Figure S1. Gas chromatography-mass spectrometry (GC-MS) chromatogram of the essential oil from the leaves of *P. aduncum* collected in the spring season.

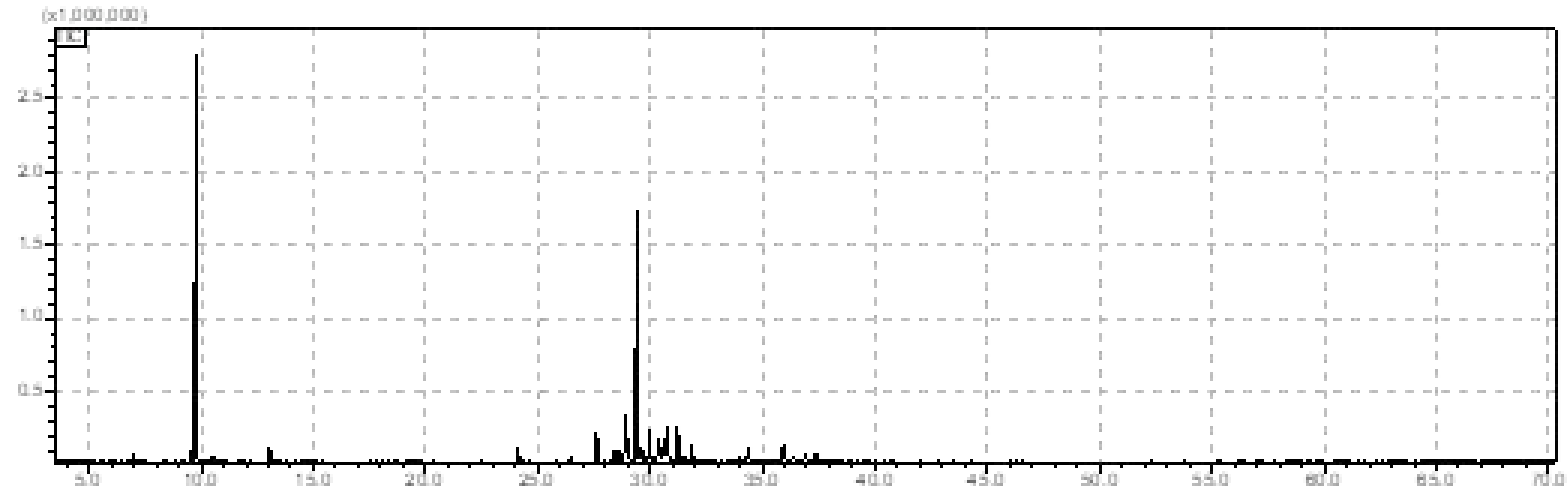

Figure S2. Gas chromatography-mass spectrometry (GC-MS) chromatogram of the essential oil from the leaves of *P. amalago* collected in the spring season.

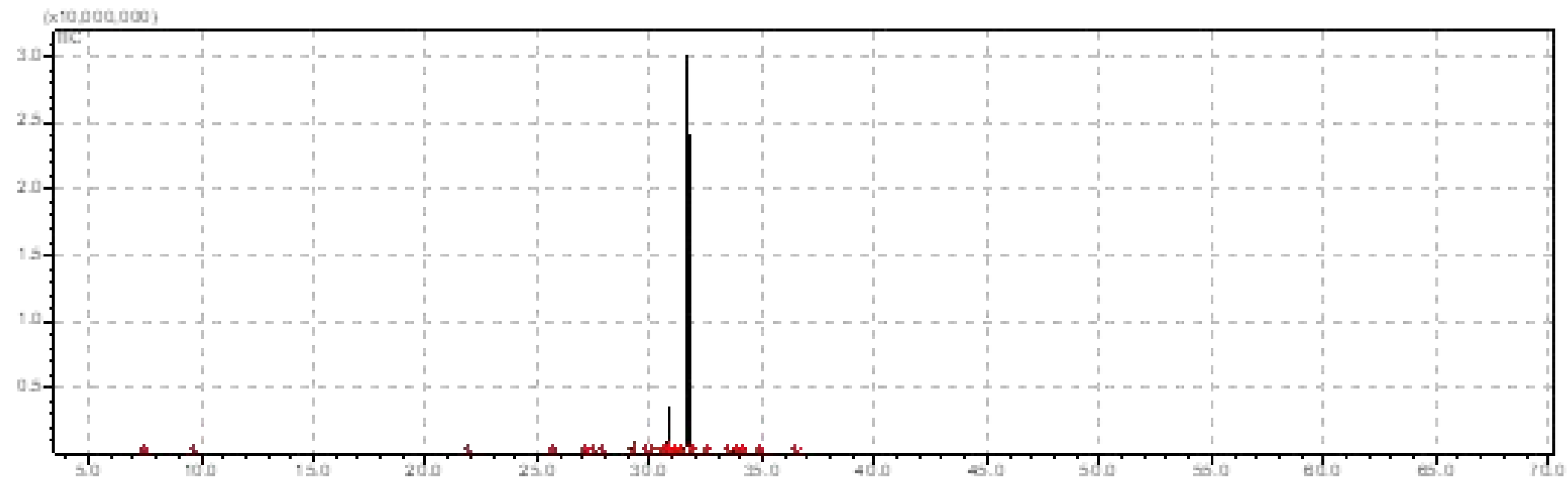

Figure S3. Gas chromatography-mass spectrometry (GC-MS) chromatogram of the essential oil from the leaves of *P. caldense* collected in the spring and summer seasons.

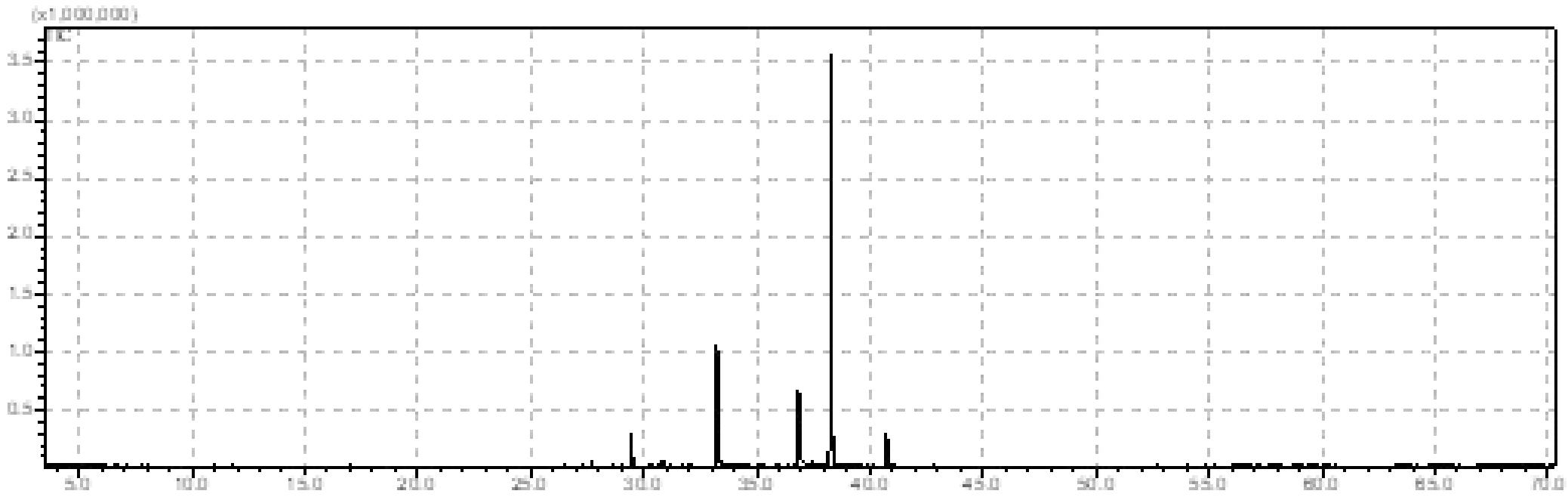

Figure S4. Gas chromatography-mass spectrometry (GC-MS) chromatogram of the essential oil from the leaves of *P. mikanianum* collected in the winter season.

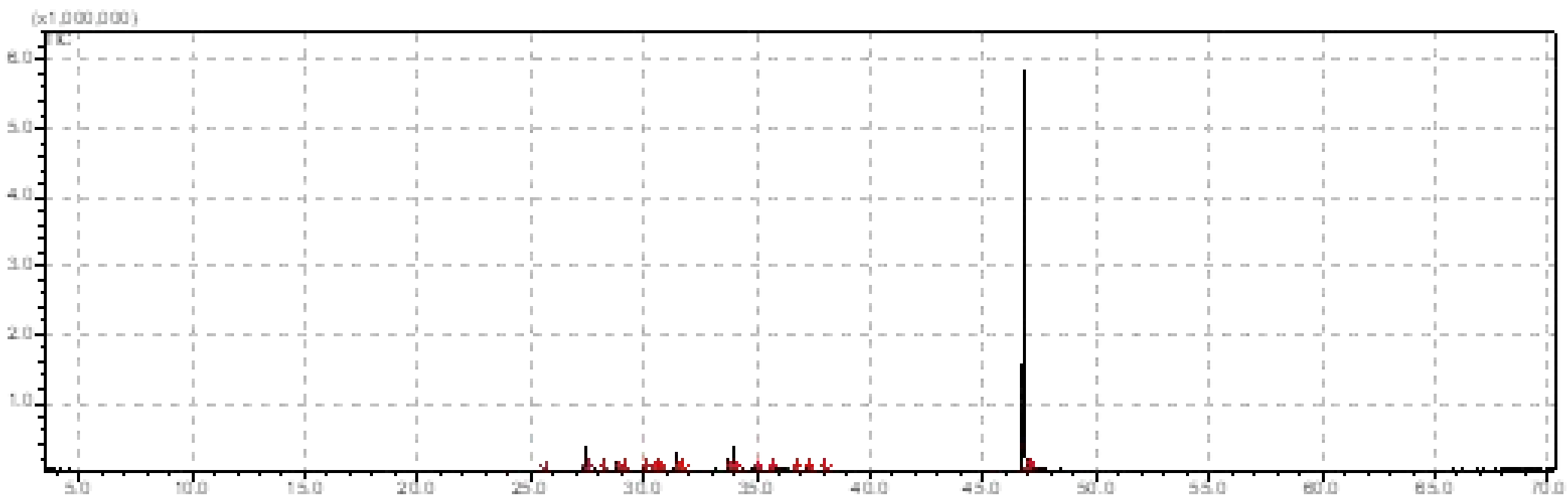

Figure S5. Gas chromatography-mass spectrometry (GC-MS) chromatogram of the essential oil from the leaves of *P. mollicomum* collected in the summer season.

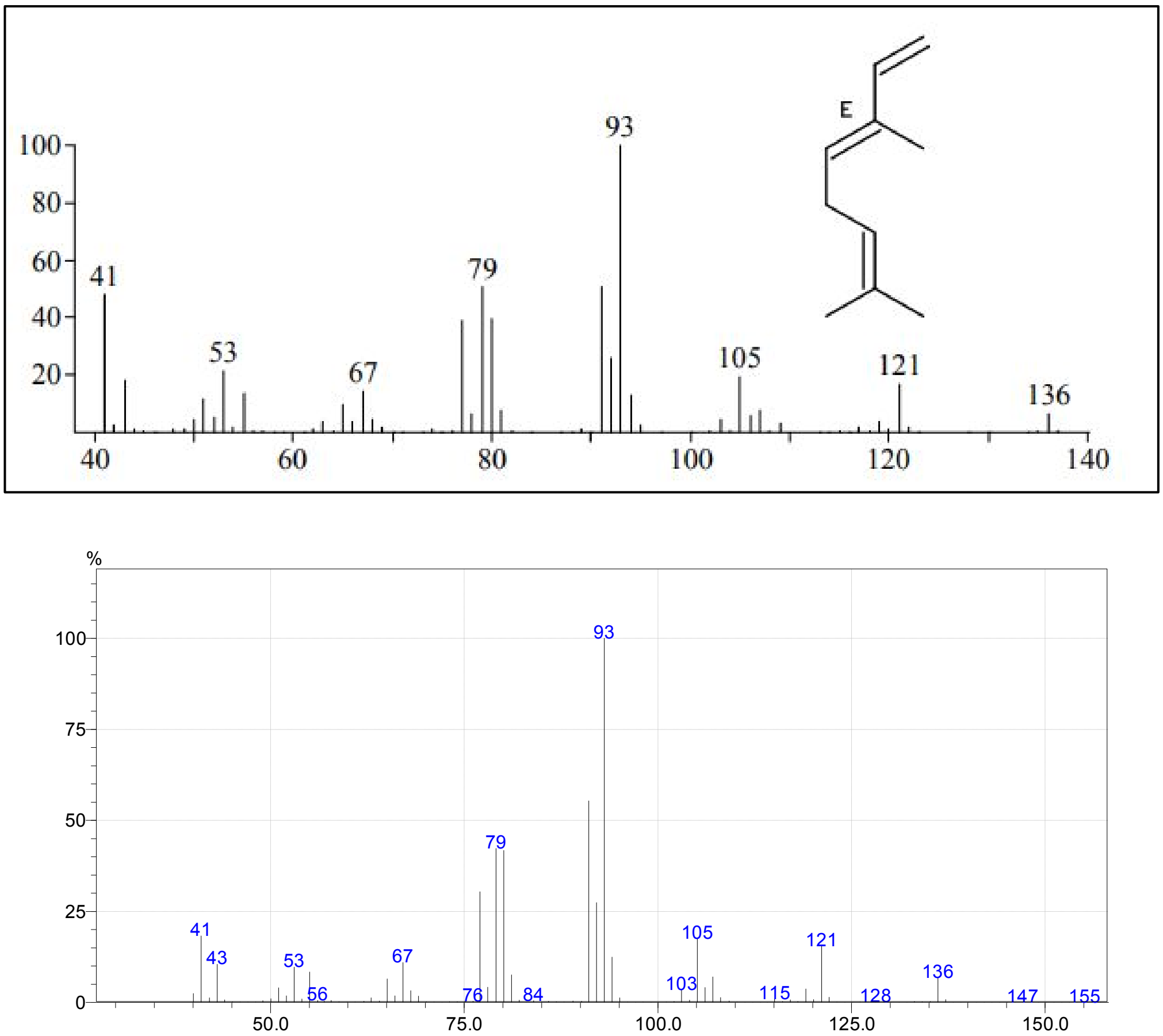

Figure S6. Comparison between the reference mass spectrum of *(E)*-β-ocimene reported by Adams[51] (top) and the experimental GC–MS mass spectrum of the constituent (bottom). The agreement of the major fragment ions, combined with the arithmetic retention index (AI) data, was used to support the identification of the compound in the essential oil of *P. aduncum*.

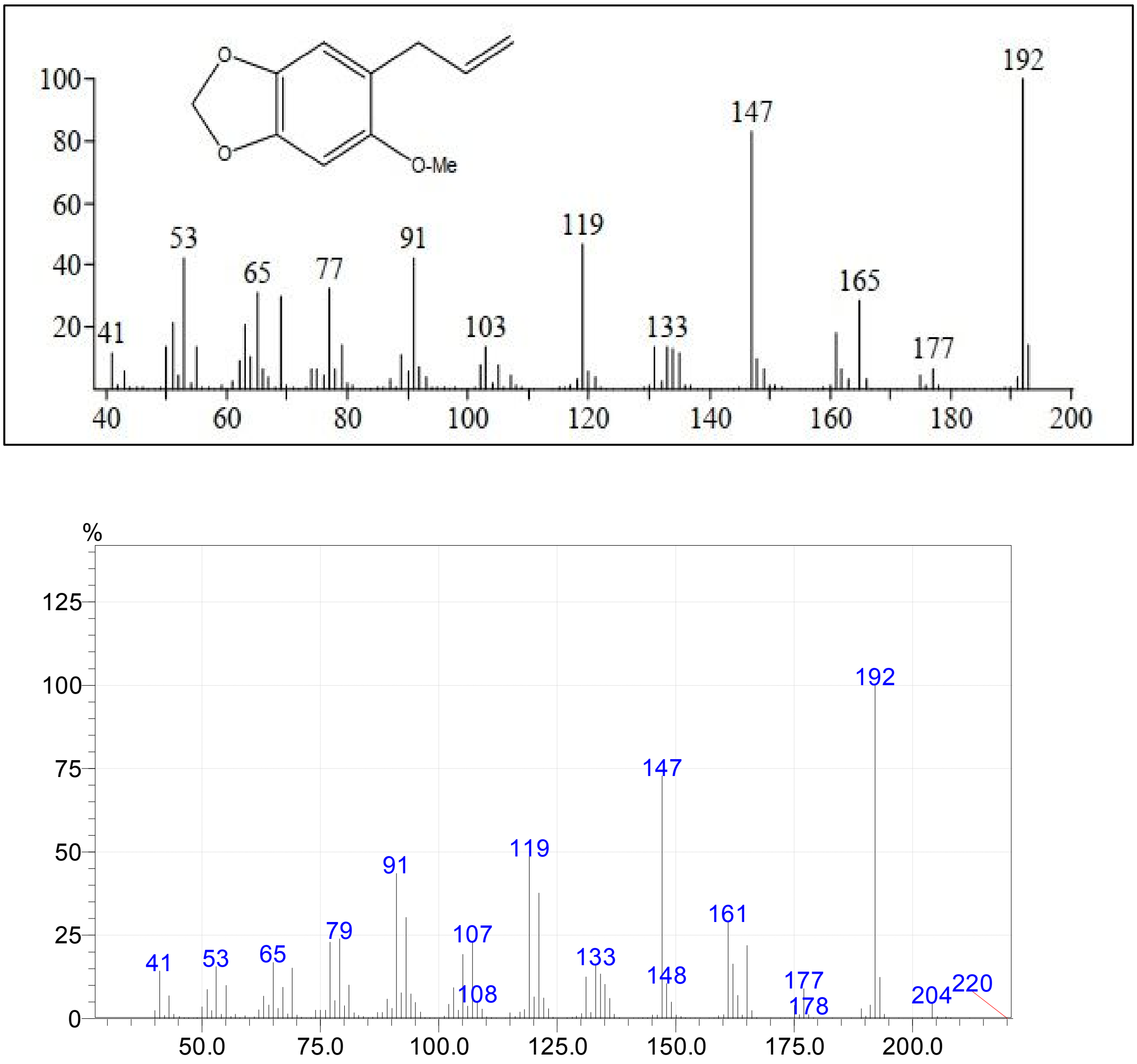

Figure S7. Comparison between the reference mass spectrum of asaricin reported by Adams[51] (top) and the experimental GC–MS mass spectrum of the constituent (bottom). The agreement of the major fragment ions, combined with the arithmetic retention index (AI) data, was used to support the identification of the compound in the essential oil of *P. aduncum*.

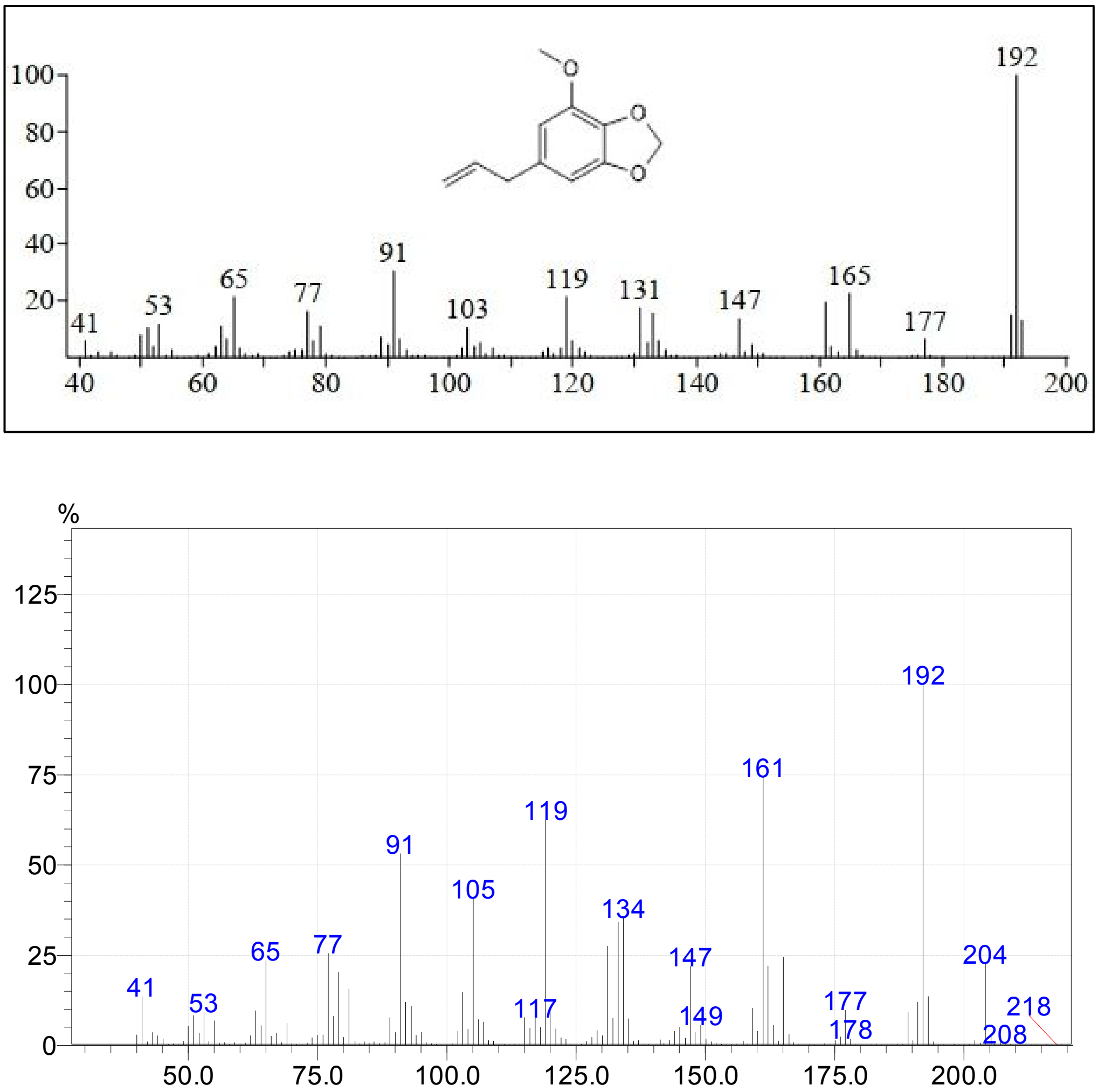

Figure S8. Comparison between the reference mass spectrum of myristicin reported by Adams[51] (top) and the experimental GC–MS mass spectrum of the constituent (bottom). The agreement of the major fragment ions, combined with the arithmetic retention index (AI) data, was used to support the identification of the compound in the essential oil of *P. aduncum* and *P. caldense*.

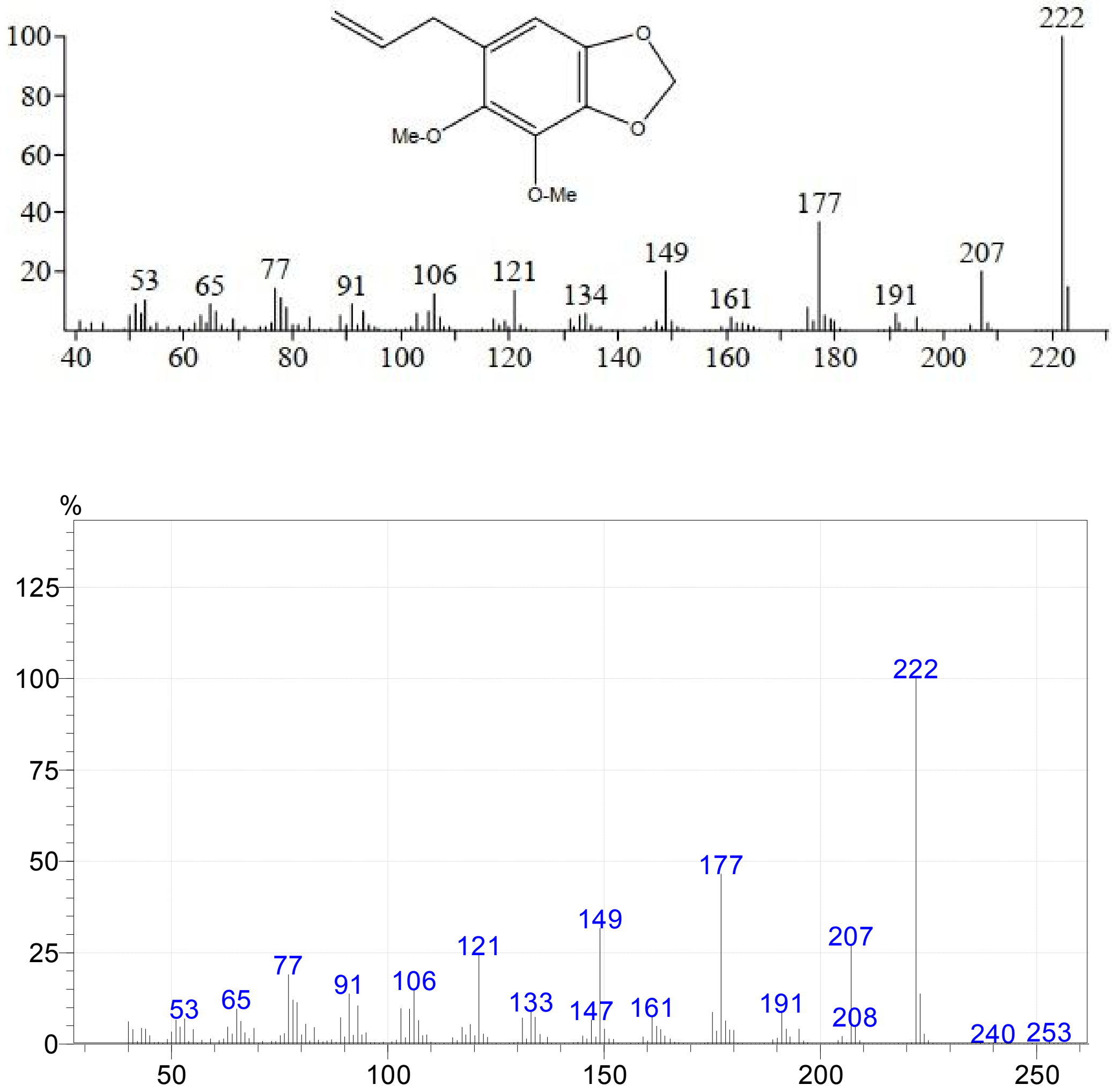

Figure S9. Comparison between the reference mass spectrum of dillapiole reported by Adams[51] (top) and the experimental GC–MS mass spectrum of the constituent (bottom). The agreement of the major fragment ions, combined with the arithmetic retention index (AI) data, was used to support the identification of the compound in the essential oil of *P. aduncum* and *P. caldense*.

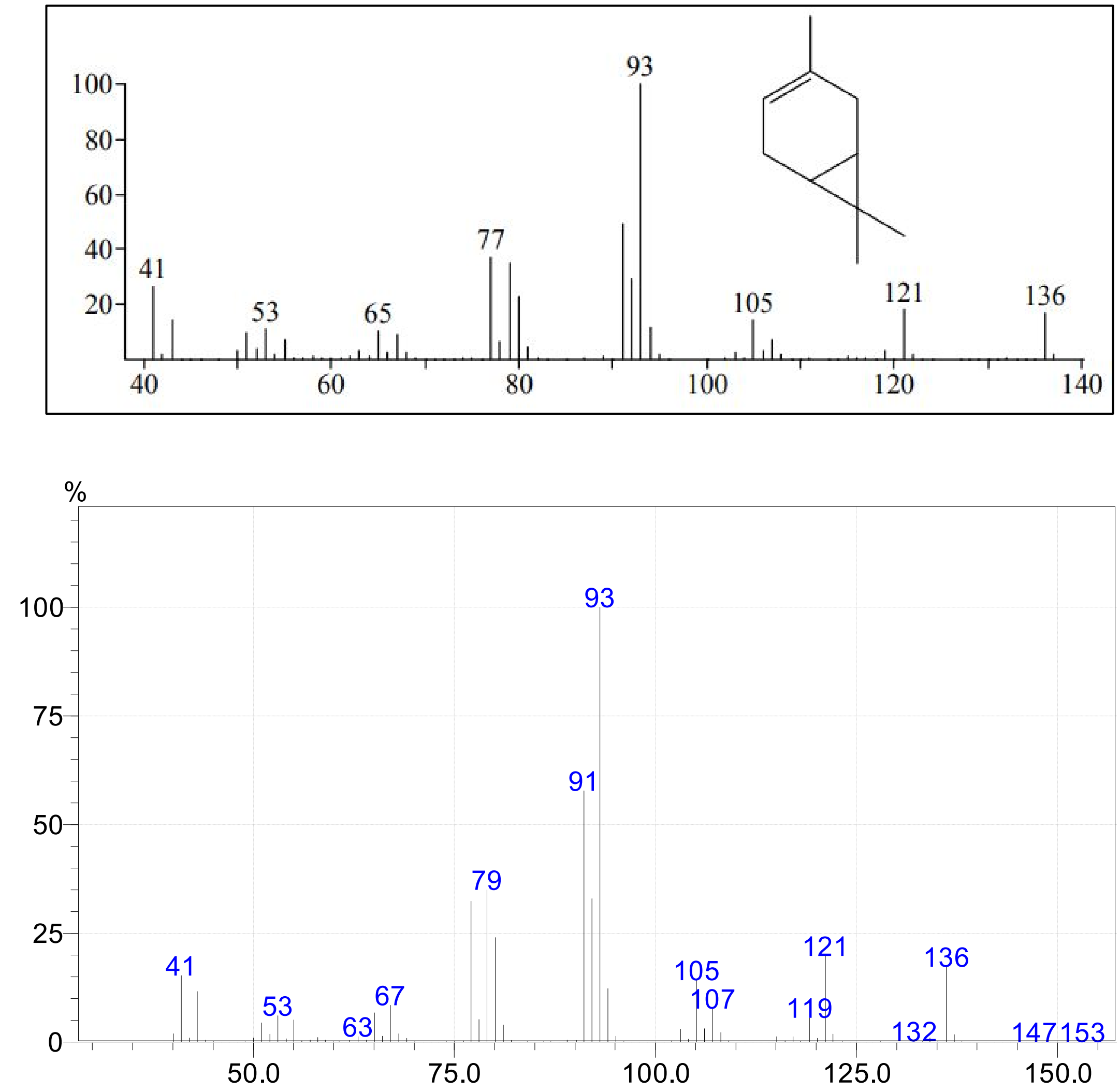

Figure S10. Comparison between the reference mass spectrum of δ 3-carene reported by Adams[51] (top) and the experimental GC–MS mass spectrum of the constituent (bottom). The agreement of the major fragment ions, combined with the arithmetic retention index (AI) data, was used to support the identification of the compound in the essential oil of *P. amalago*.

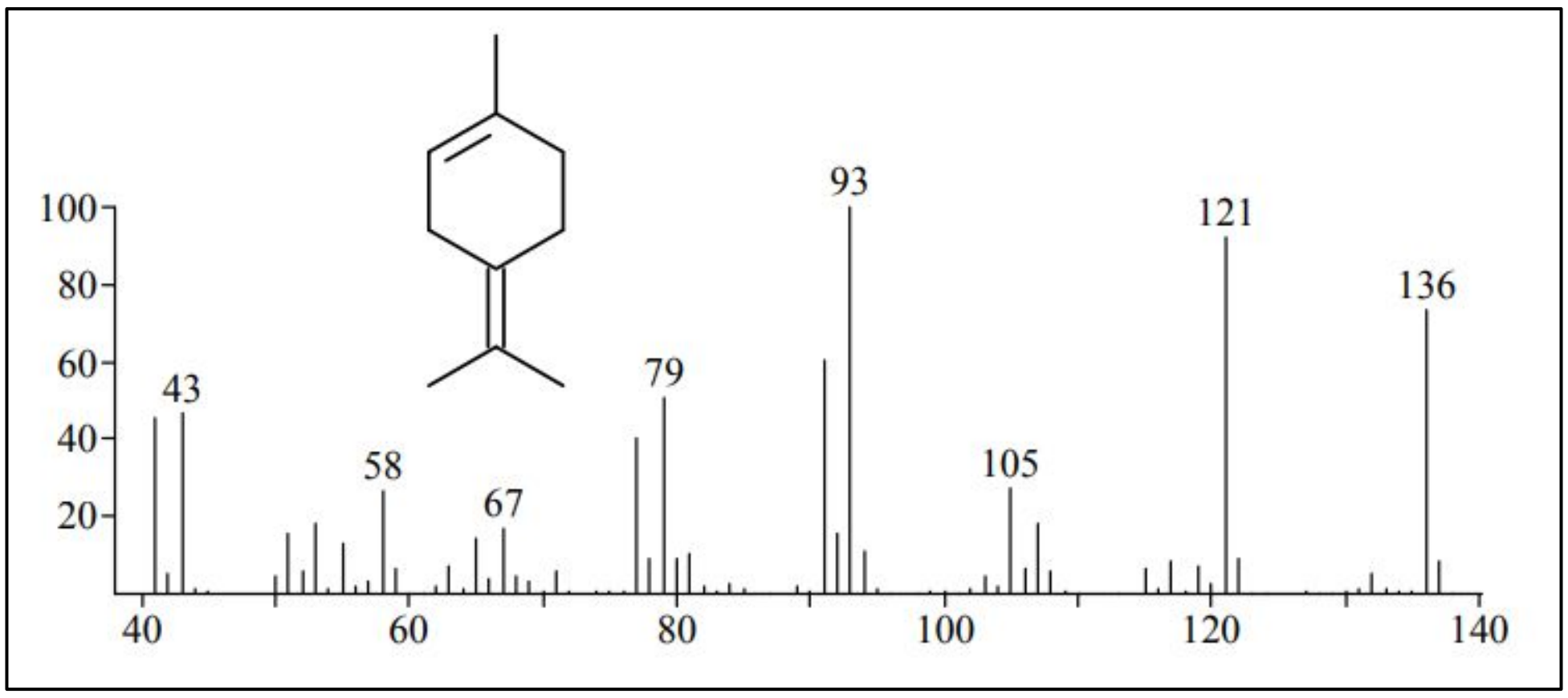

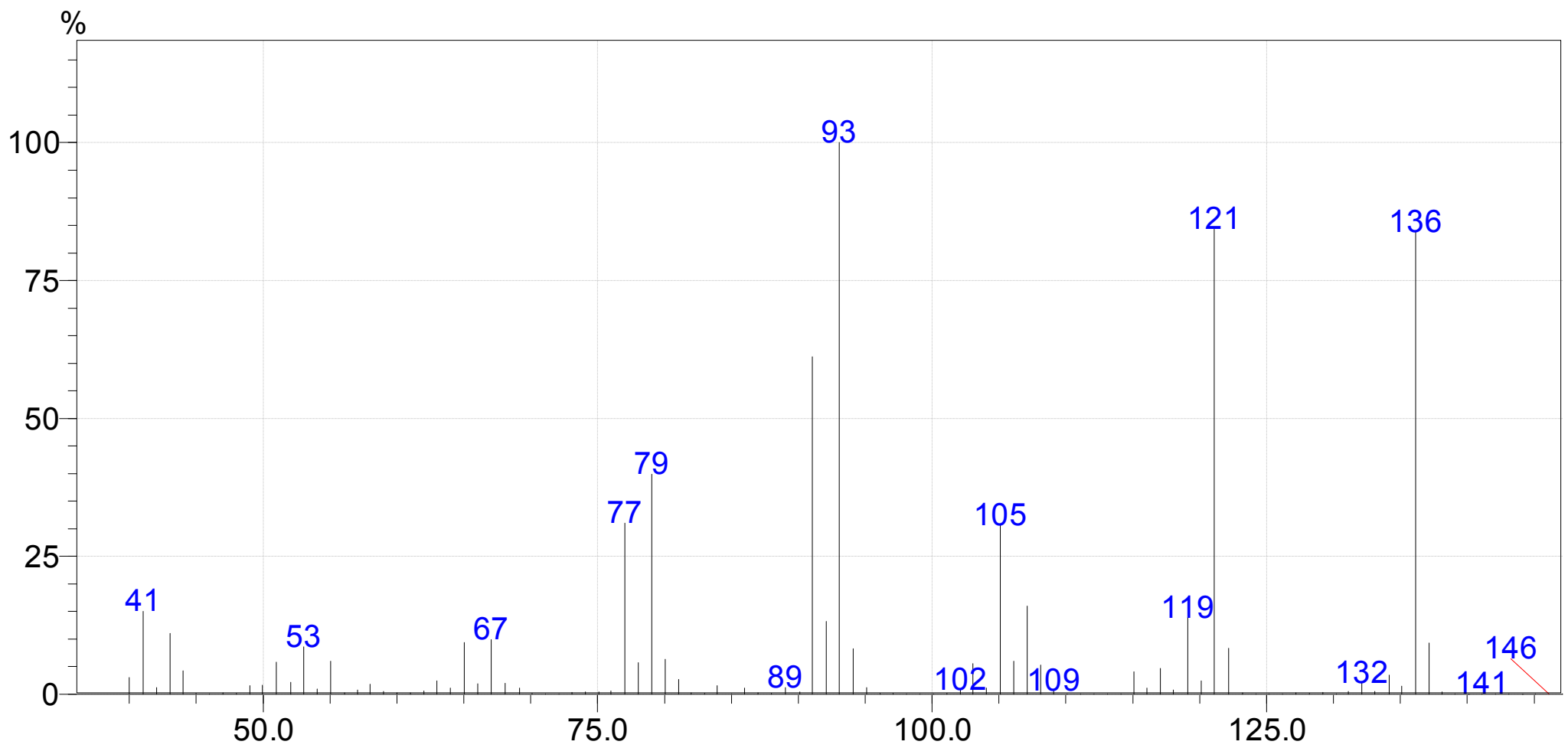

Figure S11. Comparison between the reference mass spectrum of terpinolene reported by Adams[51] (top) and the experimental GC–MS mass spectrum of the constituent (bottom). The agreement of the major fragment ions, combined with the arithmetic retention index (AI) data, was used to support the identification of the compound in the essential oil of *P. amalago*.

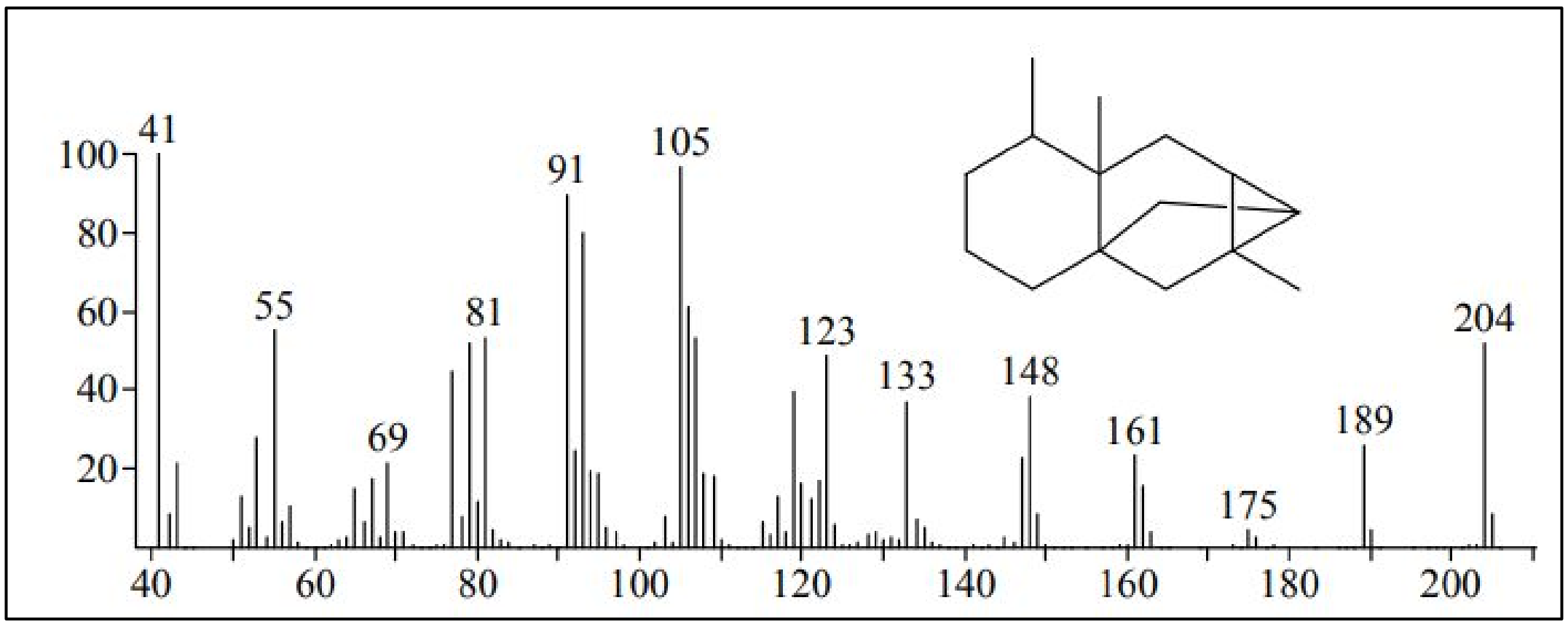

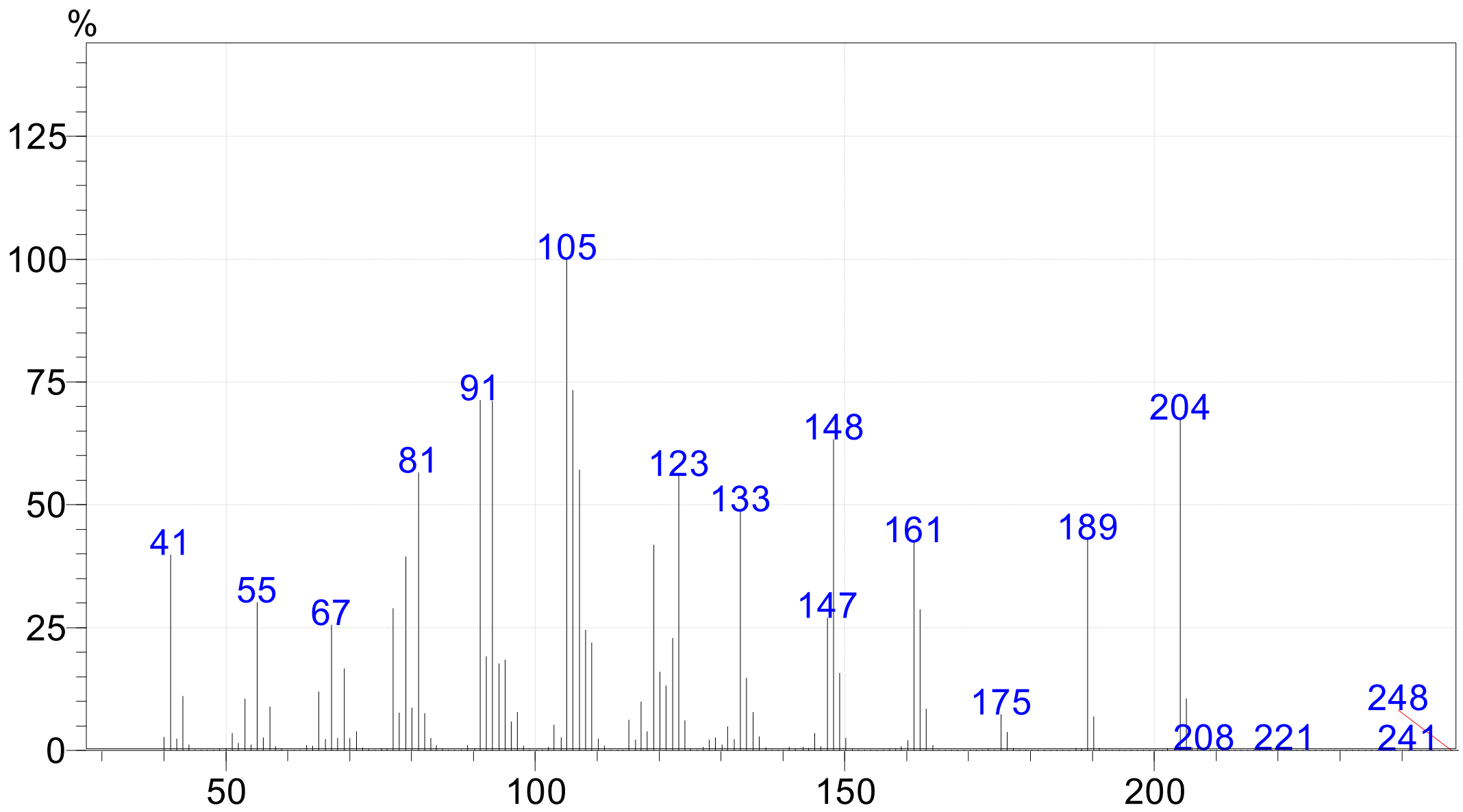

Figure S12. Comparison between the reference mass spectrum of ishwarane reported by Adams[51] (top) and the experimental GC–MS mass spectrum of the constituent (bottom). The agreement of the major fragment ions, combined with the arithmetic retention index (AI) data, was used to support the identification of the compound in the essential oil of *P. amalago* and *P. mikanianum*.

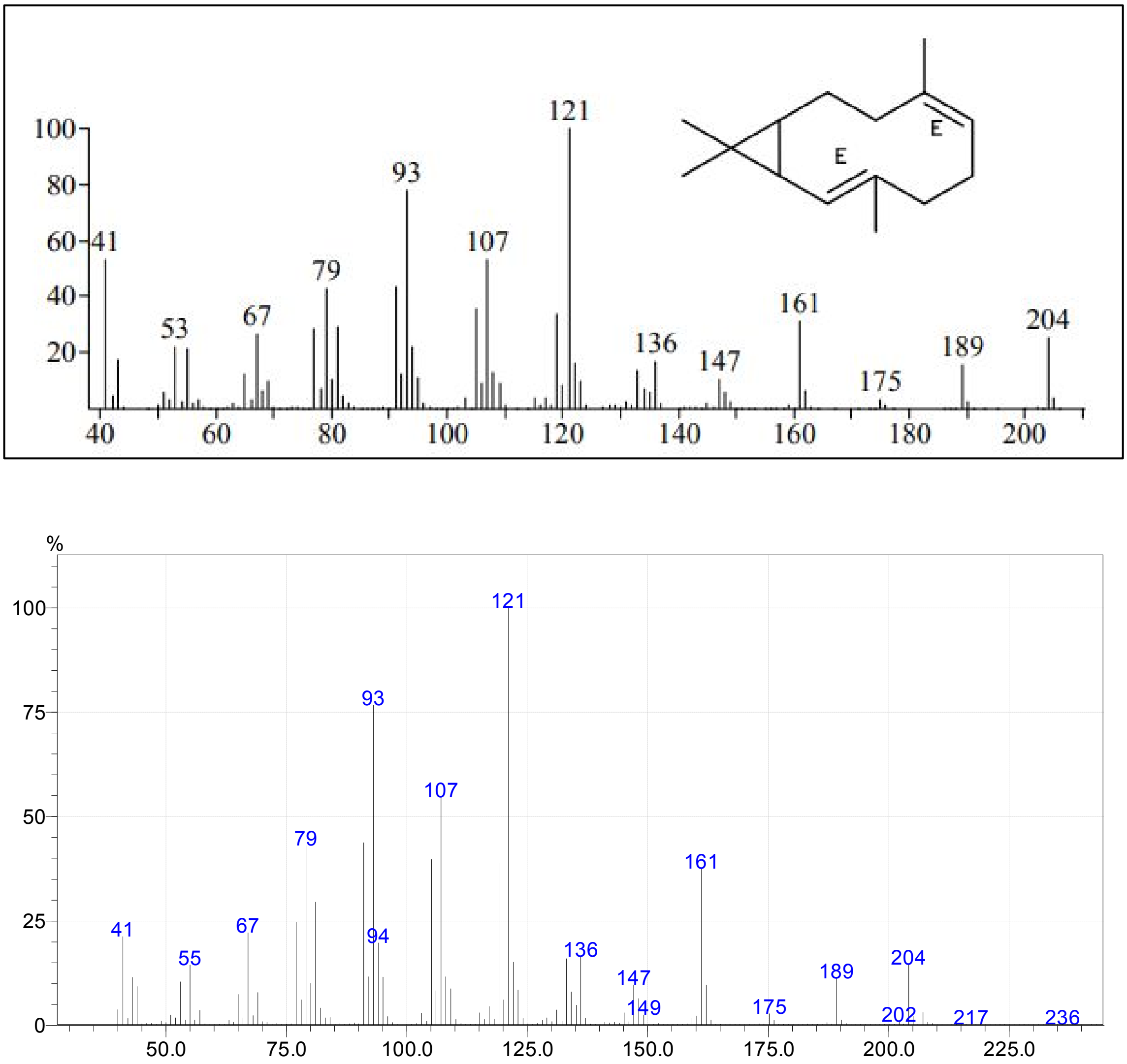

Figure S13. Comparison between the reference mass spectrum of bicyclogermacrene reported by Adams[51] (top) and the experimental GC–MS mass spectrum of the constituent (bottom). The agreement of the major fragment ions, combined with the arithmetic retention index (AI) data, was used to support the identification of the compound in the essential oil of *P. amalago*.

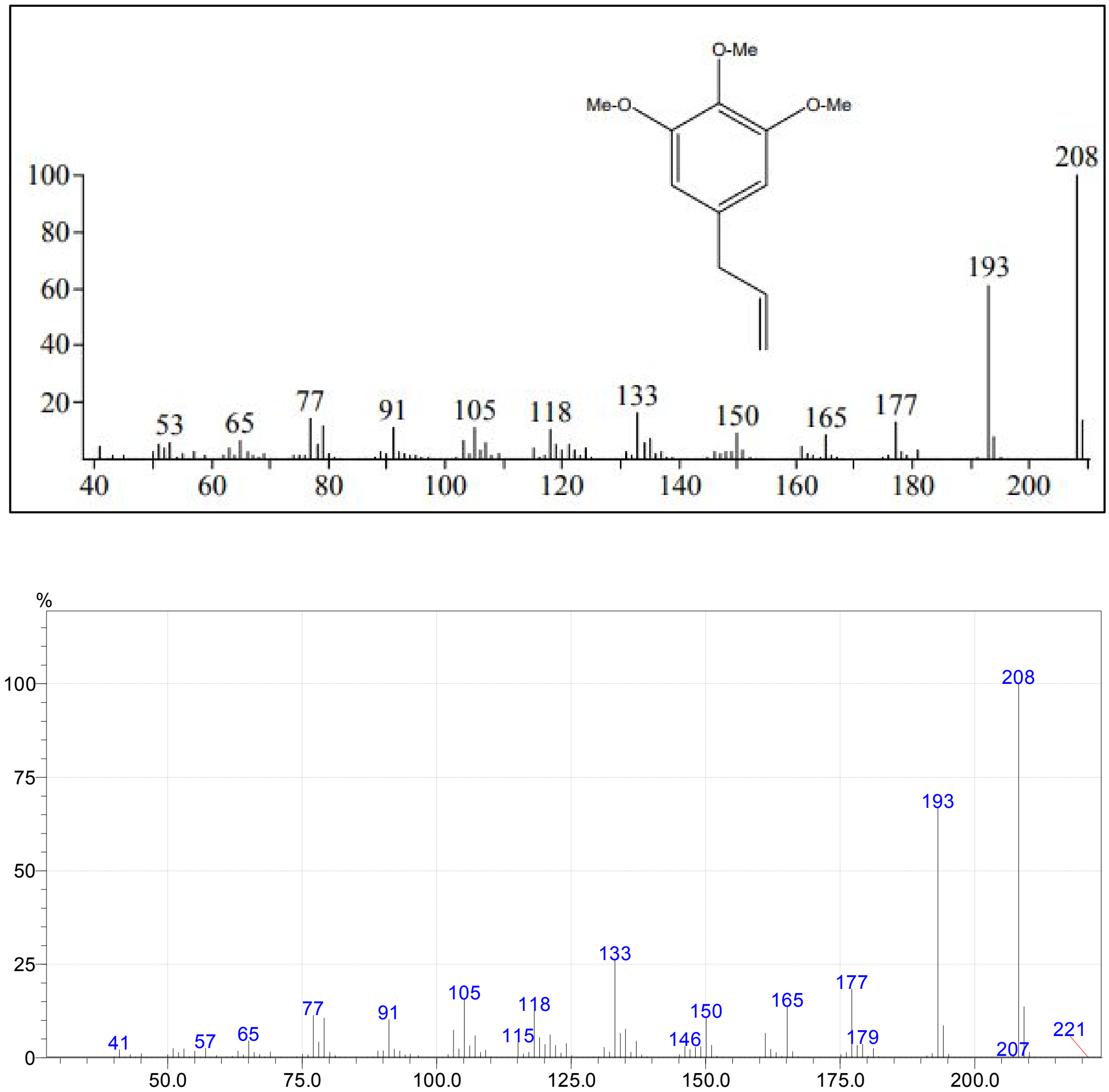

Figure S14. Comparison between the reference mass spectrum of elemicin reported by Adams[51] (top) and the experimental GC–MS mass spectrum of the constituent (bottom). The agreement of the major fragment ions, combined with the arithmetic retention index (AI) data, was used to support the identification of the compound in the essential oil of *P. mikanianum*.

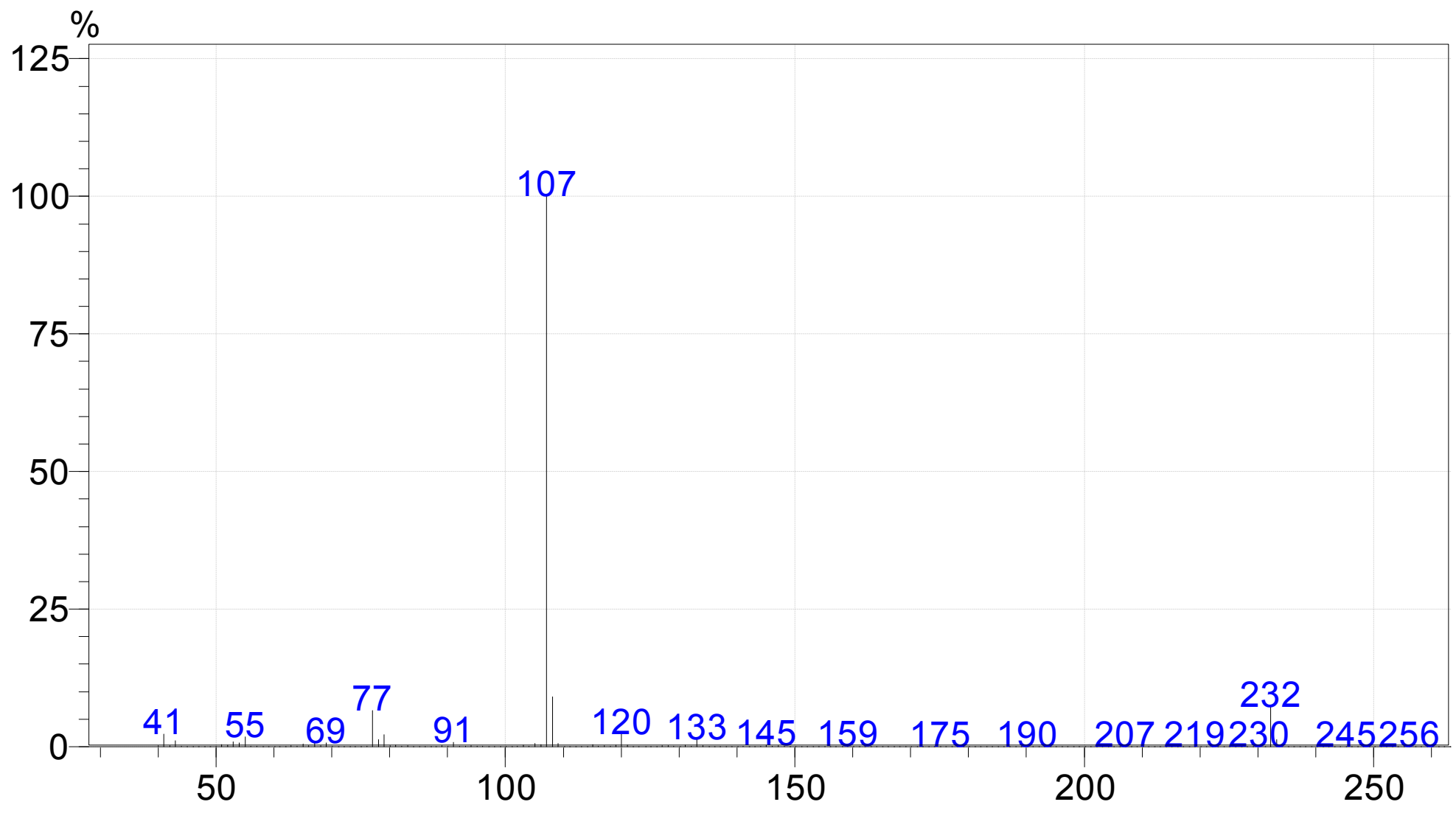

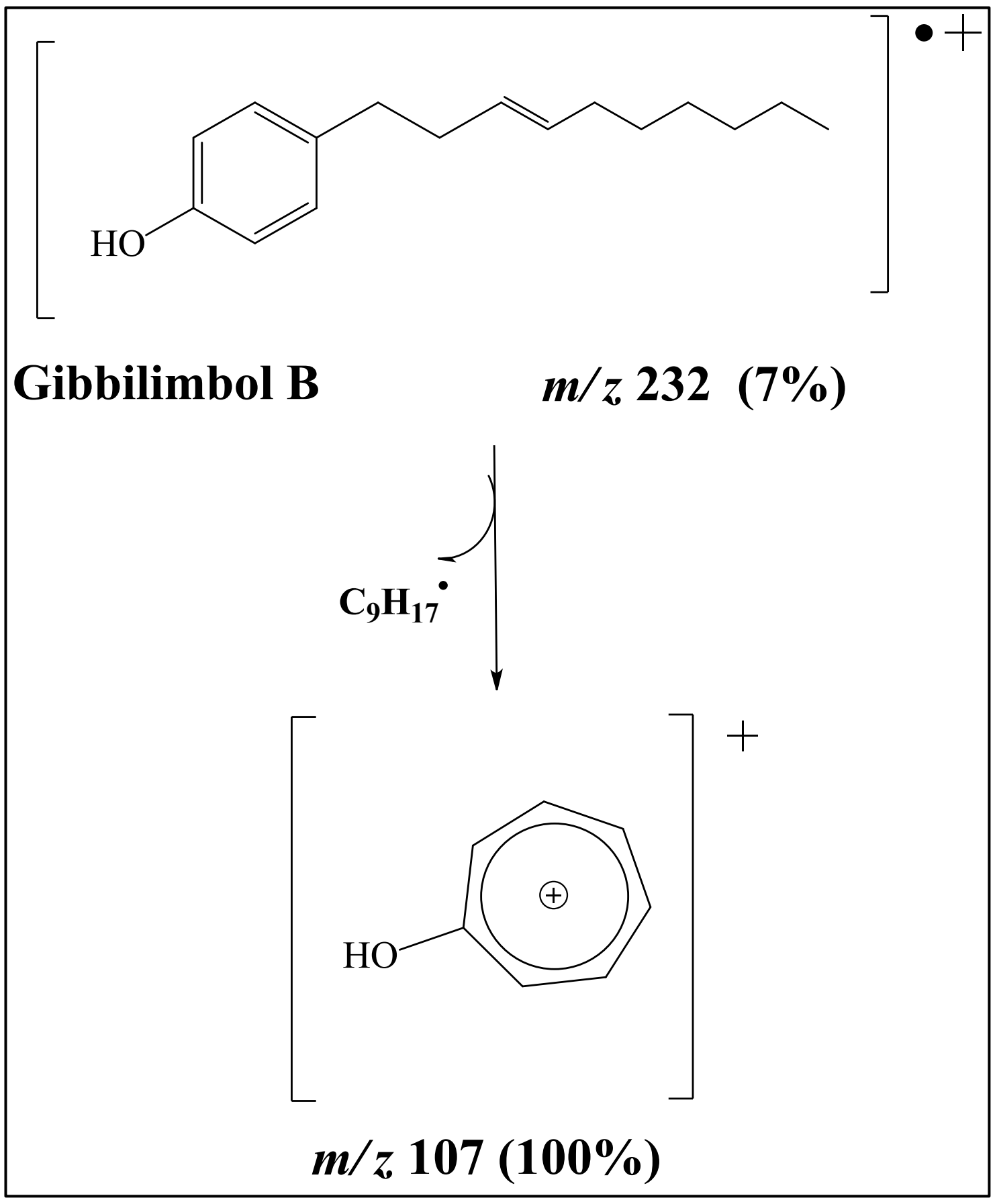

Figure S15. Mass spectrum and proposed fragmentation pathway of gibbilimbol B identified as the major constituent of the essential oil of *Piper mollicomum*. The molecular ion is observed at *m/z* 232, while the base peak at *m/z* 107 is proposed to

result from cleavage of the alkyl side chain, with loss of a $C_9H_{17}^{\bullet}$ radical and formation of a resonance-stabilized aromatic cation.

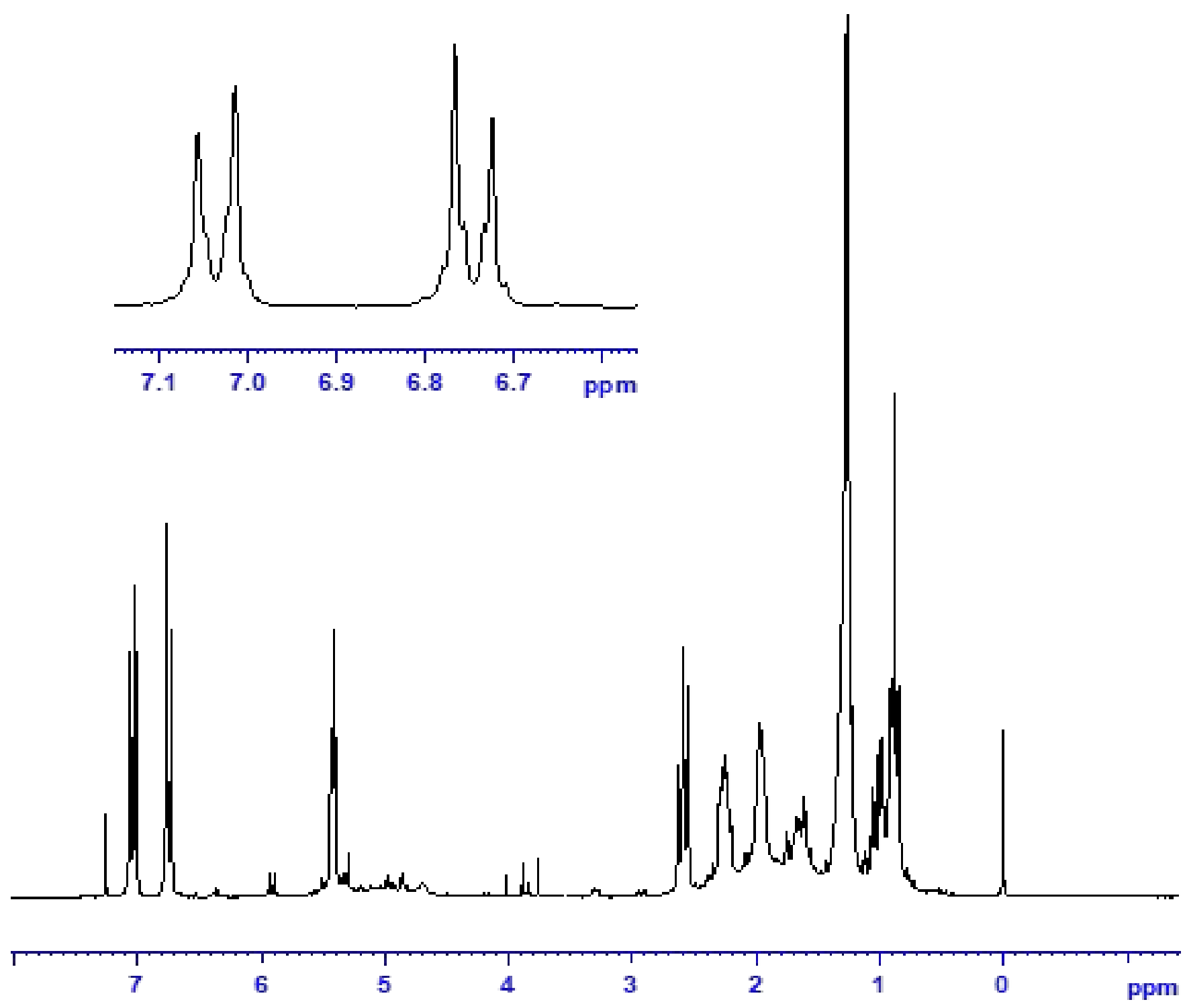

Figure S16. ^{1}H NMR spectrum (200 MHz, $CDCl_3$) of the crude essential oil of *Piper mollicomum*, showing signals attributed to gibbilimbol B, its major constituent.

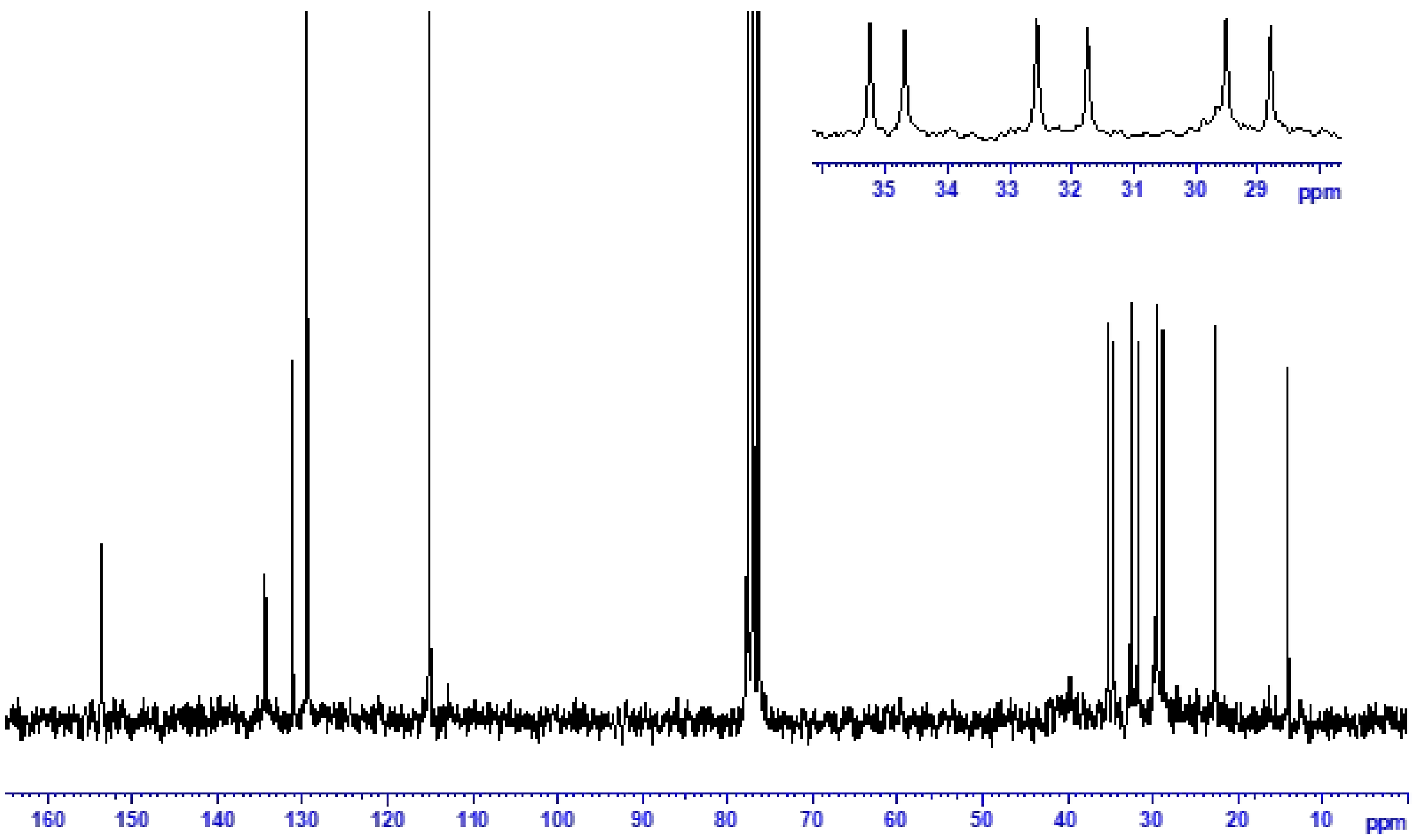

Figure S17. ^{13}C NMR spectrum (50 MHz, $CDCl_3$) of the crude essential oil of *Piper mollicomum,* showing signals attributed to gibbilimbol B, its major constituent.

Table S1- Identification of the constituents of essential oils from *Piper* species across different seasons: *P. aduncum*; *P. amalago*, *P. caldense*; *P. mikanianum; P. mollicomum*; Seasons: Spring (S); Summer (SM); Autumn (A); Winter (W)

Species				*Piper aduncum*				*Piper amalago*				*Piper caldense*				*Piper mikanianum*				*Piper mollicomum*			
Seasons				S	SM	A	W	S	SM	A	W	S	SM	A	W	S	SM	A	W	S	SM	A	W
Nº	AI[1]	AI[2]	Identified compound	(% ±SD)	(% ±SD)	(% ±SD)	(% ±SD)	(% ±SD)	(% ±SD)	(% ±SD)	(% ±SD)	(% ±SD)	(% ±SD)	(% ±SD)	(% ±SD)	(% ±SD)	(% ±SD)	(% ±SD)	(% ±SD)	(% ±SD)	(% ±SD)	(% ±SD)	(% ±SD)
1	932	932	*α*-pinene	1.87 ± 0.00	2.68 ± 0.21	0.52 ± 0.06	2.08 ± 0.81	0.72 ± 0.17	0.11 ± 0.01	0.62 ± 0.11	0.50 ± 0.07	0.34 ± 0.03	0.09 ± 0.01	2.52 ± 0.00	-	-	-	-	-	0.82 ± 0.04	-	0.29 ± 0.01	0.42 ± 0.05
2	943	946	camphene	-	-	-	-	-	-	-	-	0.18 ± 0.02	0.19 ± 0.02	0.48 ± 0.00	0.16 ± 0.04	-	-	-	-	-	-	-	-
3	972	974	β-pinene	0.80 ± 0.01	0.85 ± 0.13	0.30 ± 0.04	0.55 ± 0.07	0.19 ± 0.05	0.18 ± 0.03	-	0.17 ± 0.01	0.13 ± 0.01	-	1.14 ± 0.00	-	-	-	-	-	1.25 ± 0.37	-	0.24 ± 0.03	0.68 ± 0.07
4	991	988	myrcene	-	-	-	-	-	-	-	-	-	-	-	-	-	-	-	-	2.56 ± 1.12	-	-	0.41 ± 0.05
5	1004	1002	α-phellandrene	0.49 ± 0.00	0.82 ± 0.08	-	0.33 ± 0.02	1.18 ± 0.52	0.21 ± 0.03	1.20 ± 0.07	0.72 ± 0.02	-	-	-	-	-	-	-	-	-	-	-	-
6	1009	1008	δ-3-carene	-	-	-	-	33.93 ± 8.48	9.32 ± 0.06	31.90 ± 4.35	26.01 ± 4.62	0.12 ± 0.01	0.19 ± 0.06	0.27 ± 0.00	-	-	-	-	-	14.43 ± 4.28	-	-	1.89 ± 0.20
7	1012	1014	α-terpinene	0.77 ± 0.09	1.43 ± 0.05	-	0.58 ± 0.06	0.27 ± 0.06	-	-	-	-	-	-	-	-	-	-	-	-	-	-	-
8	1024	1020	p-cymene	0.66 ± 0.06	1.48 ± 0.03	-	0.55 ± 0.11	0.35 ± 0.06	-	-	0.80 ± 0.09	-	-	-	-	-	-	-	-	2.69 ± 0.52	-	-	0.47 ± 0.04
9	1027	1024	limonene	-	-	-	-	1.01 ± 0.33	-	-	0.30 ± 0.05	0.09 ± 0.00	-	0.45 ± 0.00	-	-	-	-	-	-	-	-	-
10	1028	1025	β-phellandrene	1.90 ± 0.00	3.16 ± 0.06	-	1.67 ± 0.03	-	-	-	-	-	-	-	-	-	-	-	-	10.55 ± 2.59	-	-	1.90 ± 0.49
11	1047	1044	(E)-β-ocimene	1.19 ± 0.00	-	-	-	-	-	-	-	0.16 ± 0.00	-	0.55 ± 0.00	-	-	-	-	-	-	-	-	-
12	1058	1054	γ-terpinene	3.36 ± 0.11	5.29 ± 0.65	-	1.79 ± 0.03	0.44 ± 0.26	-	-	1.26 ± 0.19	-	-	-	-	-	-	-	-	-	-	-	-
13	1088	1086	terpinolene	0.65 ± 0.03	0.67 ± 0.04	-	-	-	-	-	0.22 ± 0.02	-	-	-	-	-	-	-	-	-	-	-	-

14	1090	1086	p-mentha-2,4(8)-diene	-	-	-	-	2.61 ± 0.84	-	2.83 ± 0.28	2.36 ± 0.69	-	-	-	-	-	-	-	-	-	-	-	-
15	1178	1174	terpinen-4-ol	1.10 ± 0.51	6.19 ± 0.83	2.99 ± 0.26	2.96 ± 0.00	-	-	-	-	-	-	-	-	-	-	-	-	-	-	-	-
16	1255	1249	piperitone	2.98 ± 1.53	11.13 ± 3.13	8.51 ± 0.06	8.90 ± 0.23	-	-	-	-	-	-	-	-	-	-	-	-	-	-	-	-
17	1338	1335	*δ*-elemene	-	-	-	-	1.53 ± 0.00	3.96 ± 0.00	3.02 ± 0.24	-	-	-	-	-	-	-	-	-	-	-	-	-
18	1345	1345	*α*-cubebene	-	-	-	-	-	-	-	-	0.20 ± 0.05	0.12 ± 0.00	0.15 ± 0.00	0.15 ± 0.08	-	-	-	-	-	-	-	-
19	1364	1369	cyclosativene	-	-	-	-	-	-	-	-	0.49 ± 0.09	-	0.24 ± 0.00	-	-	-	-	-	0.35 ± 0.02	-	0.12 ± 0.02	0.21 ± 0.18
20	1365	1373	*α*-ylangene	-	-	-	-	-	-	-	-	0.56 ± 0.12	-	0.35 ± 0.00	-	-	-	-	-	-	-	-	-
21	1376	1374	*α*-copaene	0.52 ± 0.00	0.40 ± 0.01	0.47 ± 0.06	0.60 ± 0.06	-	-	0.14 ± 0.00	1.13 ± 0.50	2.80 ± 0.52	0.31 ± 0.04	2.79 ± 0.00	0.71 ± 0.22	-	-	-	-	1.91 ± 0.11	0.65 ± 0.14	0.51 ± 0.07	2.57 ± 0.52
22	1392	1389	*β*-elemene	-	-	-	-	1.09 ± 0.05	9.09 ± 0.99	1.33 ± 0.11	2.50 ± 0.71	0.16 ± 0.05	-	0.10 ± 0.00	-	1.63 ± 0.21	0.40 ± 0.15	0.38 ± 0.12	0.33 ± 0.07	-	-	-	-
23	1405	1400	siberene	-	-	-	-	-	-	-	-	-	-	-	-	-	-	-	-	0.22 ± 0.07	-	1.03 ± 0.14	0.16 ± 0.04
24	1409	1409	(*Z*)-caryophyllene	-	-	-	-	-	-	-	-	-	-	-	-	0.60 ± 0.15	0.75 ± 0.11	0.26 ± 0.07	0.76 ± 0.07	-	-	-	-
25	1410	1409	*α*-gurjunene	0.28 ± 0.00	-	-	-	-	-	-	-	1.19 ± 0.22	0.39 ± 0.06	2.61 ± 0.00	0.75 ± 0.24	-	-	-	-	-	-	-	-
26	1420	1417	(*E*)-caryophyllene	3.04 ± 0.87	1.90 ± 0.24	1.31 ± 0.07	2.18 ± 0.09	1.96 ± 0.46	4.17 ± 0.75	1.71 ± 0.20	0.82 ± 0.05	5.03 ± 0.67	0.55 ± 0.04	3.69 ± 0.00	1.29 ± 0.42	0.79 ± 0.08	1.22 ± 0.14	0.54 ± 0.10	1.04 ± 0.05	16.20 ± 1.80	4.34 ± 0.37	3.29 ± 0.45	16.58 ± 3.38
27	1430	1430	*β*-copaene	0.22 ± 0.03	0.21 ± 0.03	1.24 ± 0.29	2.45 ± 0.15	-	-	-	-	1.11 ± 0.21	0.35 ± 0.02	0.84 ± 0.00	0.59 ± 0.23	-	-	-	-	0.63 ± 0.04	0.12 ± 0.07	0.60 ± 0.10	0.71 ± 0.08
28	1431	1432	*α*-*trans*-bergamotene	-	-	-	-	-	-	-	-	0.26 ± 0.06	-	0.29 ± 0.00	-	-	-	-	-	-	-	-	-
29	1440	1439	aromadendrene	0.50 ± 0.06	0.18 ± 0.03	-	-	0.64 ± 0.09	-	-	0.95 ± 0.35	0.89 ± 0.15	0.15 ± 0.05	1.90 ± 0.00	0.56 ± 0.19	-	-	-	-	2.11 ± 0.22	0.70 ± 0.16	0.85 ± 0.16	1.80 ± 0.24
30	1444	1442	guaia-6,9-diene	-	-	-	-	0.84 ± 0.04	-	0.56 ± 0.02	0.61 ± 0.19	0.20 ± 0.04	-	0.33 ± 0.00	0.14 ± 0.02	-	-	-	-	-	-	-	-
31	1446	1449	spirolepechinene	-	-	-	-	-	-	-	-	-	-	-	-	-	-	-	-	-	0.11 ± 0.06	0.36 ± 0.00	0.29 ± 0.03

32	1450	1448	*cis*-muurola-3,5-diene	-	-	-	-	0.34 ± 0.04	-	0.26 ± 0.00	4.44 ± 1.47	0.37 ± 0.09	-	0.26 ± 0.00	0.37 ± 0.12	-	-	-	-	-	-	-	-
33	1452	1452	*trans*-muurola-3,5-diene	-	-	-	-	3.99 ± 0.61	7.32 ± 0.61	2.92 ± 0.01	0.29 ± 0.05	2.26 ± 0.02	0.23 ± 0.03	1.50 ± 0.00	0.45 ± 0.06	-	-	-	-	-	-	-	-
34	1453	1452	*α*-humulene	2.21 ± 0.08	1.77 ± 0.30	-	-	1.03 ± 0.12	1.13 ± 0.42	0.78 ± 0.00	-	-	-	-	-	-	-	-	-	6.39 ± 0.72	1.96 ± 0.10	0.68 ± 0.08	6.17 ± 1.06
35	1454	1454	(*E*)-*β*-farnesene	-	-	-	-	-	-	-	-	0.27 ± 0.08	0.18 ± 0.06	0.15 ± 0.00	-	-	-	-	-	-	-	-	-
36	1456	1458	*allo*-aromadendrene	0.27 ± 0.00	-	-	-	0.15 ± 0.02	-	-	0.70 ± 0.46	6.26 ± 0.45	2.04 ± 1.11	9.85 ± 0.00	3.17 ± 0.55	-	-	-	-	3.65 ± 0.45	1.16 ± 0.07	2.69 ± 0.38	3.97 ± 0.78
37	1463	1465	ishwarane	-	-	-	-	21.21 ± 1.24	26.65 ± 0.3	14.08 ± 0.98	19.9 ± 5.54	-	-	-	-	3.51 ± 0.74	5.78 ± 0.98	3.53 ± 0.67	3.42 ± 0.20	1.59 ± 0.22	-	3.41 ± 0.48	1.28 ± 0.38
38	1469	1469	*β*-acoradiene	-	-	-	-	-	1.66 ± 0.52	1.03 ± 0.04	-	-	-	-	-	-	-	-	-	-	-	-	-
39	1470	1471	dauca-5,8-diene	-	-	-	-	-	-	-	-	0.80 ± 0.17	-	0.51 ± 0.00	0.34 ± 0.10	-	-	-	-	-	-	-	-
40	1474	1475	*trans*-cardina-1(6),4-diene	0.69 ± 0.00	0.26 ± 0.04	-	0.38 ± 0.09	0.18 ± 0.02	-	0.23 ± 0.00	0.36 ± 0.03	-	-	-	-	-	-	-	-	-	-	-	-
41	1477	1478	*γ*-muurolene	-	-	-	-	1.76 ± 0.27	-	0.39 ± 0.03	1.78 ± 1.08	2.06 ± 0.21	0.37 ± 0.09	2.51 ± 0.00	0.63 ± 0.15	-	-	-	-	0.34 ± 0.08	0.18 ± 0.03	0.48 ± 0.02	0.45 ± 0.04
42	1482	1484	germacrene D	4.76 ± 0.81	1.38 ± 0.19	-	-	0.67 ± 0.09	0.95 ± 0.05	1.09 ± 0.07	0.86 ± 0.24	1.47 ± 0.16	0.68 ± 0.05	1.39 ± 0.00	0.36 ± 0.11	0.33 ± 0.15	0.34 ± 0.14	-	0.45 ± 0.07	0.19 ± 0.02	-	0.54 ± 0.00	0.23 ± 0.02
43	1484	1489	*β*-selinene	-	-	-	-	-	-	-	-	0.77 ± 0.12	-	0.77 ± 0.00	0.28 ± 0.08	-	-	-	-	0.56 ± 0.05	0.18 ± 0.03	0.45 ± 0.02	0.80 ± 0.04
44	1485	1487	*cis*-eudesma-6,11-diene	-	-	-	-	1.55 ± 0.56	-	0.57 ± 0.17	0.63 ± 0.14	-	-	-	-	-	-	-	-	-	-	-	-
45	1487	1489	aristochene	-	-	-	-	0.66 ± 0.09	1.13 ± 0.32	0.19 ± 0.08	1.64 ± 0.86	-	-	-	-	-	-	-	-	-	0.83 ± 0.13	0.37 ± 0.01	2.12 ± 0.35
46	1489	1492	*β*-cis-guaiene	-	-	-	-	-	1.74 ± 0.17	-	0.53 ± 0.05	-	-	-	-	-	-	-	-	1.37 ± 0.12	0.82 ± 0.28	2.09 ± 0.11	4.81 ± 1.75
47	1492	1493	epi-cubebol	-	-	-	-	-	-	-	-	1.88 ± 0.63	0.41 ± 0.04	3.36 ± 0.00	1.05 ± 0.21	-	-	-	-	-	-	-	-
48	1494	1493	*trans*-muurola-4(14),5-diene	-	-	-	-	1.83 ± 0.13	-	1.55 ± 0.13	0.33 ± 0.28	-	-	-	-	-	-	-	-	-	-	-	-

49	1496	1496	valencene	-	-	-	-	-	-	-	-	-	-	-	-	2.38 ± 0.67	0.13 ± 0.17	-	-	-	-	-	-
50	1497	1496	viridiflorene	-	-	-	-	4.68 ± 0.34	-	-	1.69 ± 0.16	-	-	-	-	-	0.29 ± 0.02	0.30 ± 0.05	-	-	-	-	-
51	1498	1495	asaricin	30.23 ± 1.03	18.92 ± 1.48	24.95 ± 0.13	23.90 ± 0.53	-	-	-	-	-	-	-	-	-	-	-	-	-	-	-	-
52	1499	1500	bicyclogermacrene	-	-	-	-	-	11.43 ± 1.31	9.84 ± 0.49	6.09 ± 2.53	2.07 ± 0.40	0.91 ± 0.09	4.30 ± 0.00	1.96 ± 0.32	7.89 ± 2.61	1.30 ± 0.32	0.82 ± 0.26	2.19 ± 0.28	2.72 ± 0.51	0.36 ± 0.03	2.32 ± 0.11	2.41 ± 1.21
53	1500	1502	trans-*β*-guaiene	-	-	-	-	-	-	-	-	-	-	-	-	-	-	-	-	0.74 ± 0.07	-	0.10 ± 0.01	0.54 ± 0.04
54	1501	1500	*α*-muurolene	-	-	-	-	0.55 ± 0.00	-	0.80 ± 0.00	0.63 ± 0.12	2.79 ± 0.11	0.19 ± 0.03	2.40 ± 0.00	1.82 ± 0.30	-	-	-	-	0.77 ± 0.12	0.42 ± 0.03	2.27 ± 0.09	1.10 ± 0.01
55	1502	1505	*β*-bisabolene	-	-	-	-	-	-	-	-	4.00 ± 0.77	9.18 ± 2.35	-	0.19 ± 0.03	-	-	-	-	-	-	-	-
56	1503	1505	(*E*,*E*)-*α*-farnesene	-	-	-	-	-	-	-	-	1.84 ± 0.40	1.06 ± 0.26	0.89 ± 0.00	0.16 ± 0.09	-	-	-	-	-	-	-	-
57	1510	1511	*δ*-amorphene	1.54 ± 0.17	1.22 ± 0.60	0.82 ± 0.12	0.81 ± 0.02	2.36 ± 0.37	-	2.34 ± 0.19	3.12 ± 0.82	1.51 ± 0.07	0.68 ± 0.03	1.81 ± 0.00	0.76 ± 0.02	-	-	-	-	-	-	-	-
58	1515	1513	*γ*-cardinene	-	-	-	-	-	-	-	-	-	-	-	-	-	-	-	-	0.78 ± 0.13	0.20 ± 0.03	1.67 ± 0.00	1.24 ± 0.20
59	1516	1514	cubebol	-	-	-	-	-	-	-	-	-	-	-	-	-	-	-	-	0.18 ± 0.01	3.06 ± 0.08	1.04 ± 0.01	7.19 ± 1.55
60	1522	1522	*δ*-cadinene	-	-	-	-	1.81 ± 0.08	4.49 ± 0.10	3.38 ± 0.29	-	-	-	-	-	0.43 ± 0.04	0.29 ± 0.05	-	-	-	-	-	-
61	1524	1517	myristicin	15.71 ± 2.10	10.68 ± 0.75	17.63 ± 0.30	16.68 ± 1.63	-	-	-	-	32.46 ± 4.57	74.51 ± 11.89	16.17 ± 0.00	62.3 ± 6.77	-	-	-	-	1.02 ± 0.09	1.32 ± 0.14	4.54 ± 0.13	4.59 ± 0.81
62	1530	1537	*α*-cadinene	-	-	-	-	-	3.86 ± 0.69	-	1.70 ± 0.64	-	-	-	-	-	-	-	-	-	-	-	-
63	1531	1533	*trans*-cadina-1,4-diene	-	-	-	-	-	-	-	-	0.20 ± 0.09	-	0.47 ± 0.00	0.24 ± 0.00	-	-	-	-	-	-	-	-
64	1539	1544	*α*-calacorene	-	-	-	-	-	-	-	-	1.65 ± 0.15	0.38 ± 0.19	2.22 ± 0.00	0.53 ± 0.04	-	-	-	-	0.24 ± 0.03	-	1.09 ± 0.07	0.18 ± 0.02
65	1553	1559	germacrene B	-	-	-	-	-	-	-	-	0.11 ± 0.02	-	0.22 ± 0.00	0.05	-	-	-	-	-	-	-	-
66	1558	1561	(*E*)-nerolidol	-	-	-	-	-	-	-	-	1.87 ± 0.38	0.28 ± 0.03	1.29 ± 0.00	0.75 ± 0.03	-	-	-	-	-	-	-	-

67	1560	1555	(*E*)-elemicin	-	-	-	-	-	-	-	-	-	-	-	-	21.40 ± 5.28	3.10 ± 1.09	16.80 ± 0.33	7.73 ± 0.80	-	-	-	-
68	1565	1567	palustrol	-	-	-	-	-	-	-	-	1.43 ± 0.16	0.30 ± 0.02	2.29 ± 0.00	0.86 ± 0.12	-	-	-	-	-	-	-	-
69	1569	1568	(*Z*)-elemicin	0.24 ± 0.03	0.31 ± 0.03	0.69 ± 0.18	0.67 ± 0.11	-	-	-	-	-	-	-	-	-	-	-	-	-	-	-	-
70	1576	1577	spathulenol	1.80 ± 0.10	4.59 ± 0.40	4.83 ± 0.44	3.74 ± 0.17	-	-	-	-	1.16 ± 0.12	1.17 ± 0.51	1.91 ± 0.00	3.20 ± 0.32	-	-	-	-	2.09 ± 0.24	2.19 ± 0.04	1.36 ± 0.78	4.03 ± 0.71
71	1579	1586	thujopsan-2-*α*-ol	-	-	-	-	-	-	-	-	2.47 ± 0.03	0.57 ± 0.05	-	2.02 ± 0.19	-	-	-	-	-	-	-	-
72	1584	1582	caryophyllene oxide	1.65 ± 0.05	2.71 ± 0.19	5.33 ± 0.00	3.45 ± 0.28	-	-	-	-	-	-	-	-	-	-	-	-	-	-	-	-
73	1589	1590	globulol	3.29 ± 0.27	5.43 ± 0.56	9.05 ± 0.14	4.90 ± 0.02	0.99 ± 0.05	0.40 ± 0.46	0.47 ± 0.02	0.25 ± 0.02	0.72 ± 0.01	-	1.08 ± 0.00	0.42 ± 0.07	-	-	-	-	3.84 ± 0.72	5.10 ± 0.01	1.36 ± 0.48	6.04 ± 0.08
74	1591	1592	viridiflorol	-	-	-	-	-	1.43 ± 0.11	1.43 ± 0.11	1.02 ± 0.80	-	-	-	-	-	-	-	-	0.39 ± 0.11	0.14 ± 0.09	1.00 ± 0.41	0.98 ± 0.08
75	1593	1595	cubeban-11-ol	-	-	-	-	-	-	-	-	-	-	-	-	-	-	-	-	0.27 ± 0.05	-	8.32 ± 0.16	0.24 ± 0.02
76	1596	1600	guaiol	-	-	-	-	-	-	-	-	-	-	-	-	-	-	-	-	0.31 ± 0.07	-	2.67 ± 0.06	-
77	1599	1602	ledol	-	-	-	-	-	-	-	-	3.23 ± 0.22	1.14 ± 0.31	6.95 ± 0.00	3.10 ± 0.12	-	-	-	-	0.52 ± 0.14	0.17 ± 0.03	0.41 ± 0.01	0.67 ± 0.06
78	1611	1608	humulene epoxide II	1.22 ± 0.04	1.67 ± 0.03	4.27 ± 0.23	1.87 ± 0.11	-	-	-	-	-	-	-	-	-	-	-	-	0.11 ± 0.05	0.63 ± 0.09	25.47 ± 3.82	0.11 ± 0.09
79	1623	1620	dillapiole	7.40 ± 0.97	2.87 ± 0.85	3.04 ± 0.08	7.92 ± 1.83	-	-	-	-	-	-	-	-	-	-	-	-	-	-	-	-
80	1625	1627	1-epi-cubenol	-	-	-	-	-	0.10 ± 0.14	0.08 ± 0.00	0.23 ± 0.01	1.13 ± 0.07	0.21 ± 0.01	1.52 ± 0.00	0.90 ± 0.07	-	-	-	-	-	-	-	-
81	1626	1630	*γ*-eudesmol	-	-	-	-	-	-	-	-	-	-	-	-	-	-	-	-	0.37 ± 0.10	0.49 ± 0.03	0.76 ± 0.05	0.37 ± 0.05
82	1631	1630	muurola-4,10(14)-dien-1-*β*-ol	-	-	-	-	1.36 ± 0.14	1.18 ± 0.08	1.18 ± 0.06	2.93 ± 0.58	0.58 ± 0.02	0.36 ± 0.04	0.81 ± 0.00	0.35 ± 0.01	-	-	-	-	-	-	-	-
83	1634	1640	epi-*α*-cadinol	-	-	-	-	-	-	-	-	-	-	-	-	-	-	-	-	0.25 ± 0.04	0.13 ± 0.01	1.61 ± 0.01	0.56 ± 0.05
84	1638	1640	epi-*α*-muurolol	-	-	-	-	-	-	-	-	1.66 ± 0.41	0.35 ± 0.07	3.23 ± 0.00	1.56 ± 0.11	-	-	-	-	-	-	-	-

85	1643	1644	*α*-muurolol (=torreyol)	-	-	-	-	-	-	-	-	-	-	-	-	-	-	-	-	0.93 ± 0.20	0.42 ± 0.06	3.50 ± 0.18	1.50 ± 0.39
86	1644	1645	cubenol	0.63 ± 0.14	1.38 ± 0.52	0.97 ± 0.13	0.92 ± 0.16	0.60 ± 0.03	0.77 ± 0.06	0.77 ± 0.07	1.20 ± 0.44	-	-	-	-	-	-	-	-	-	-	-	-
87	1656	1653	4,6-dimethoxy-5-vinyl-1,2-benzodioxole	-	-	-	-	-	-	-	-	-	-	-	-	17.43 ± 2.67	19.87 ± 2.41	13.07 ± 0.54	21.88 ± 1.06	-	-	-	-
88	1661	1652	*α*-cadinol	-	-	-	-	0.84 ± 0.03	1.27 ± 0.35	1.27 ± 0.51	-	1.28 ± 0.24	0.34 ± 0.03	2.51 ± 0.00	1.10 ± 0.20	-	-	-	-	0.92 ± 0.20	0.91 ± 0.24	3.01 ± 0.12	1.18 ± 0.13
89	1663	1668	14-hydroxy-9-epi-(*E*)-caryophyllene	-	-	-	-	1.03 ± 0.13	0.47 ± 0.03	0.47 ± 0.05	-	-	-	-	-	-	-	-	-	0.19 ± 0.02	0.21 ± 0.03	0.09 ± 0.13	-
90	1665	1661	*allo*himachalol	-	-	-	-	-	-	-	-	-	-	-	-	0.37 ± 0.04	0.37 ± 0.03	0.54 ± 0.02	0.41 ± 0.05	-	-	-	-
91	1671	1675	cadalene	-	-	-	-	-	-	-	-	1.07 ± 0.10	0.27 ± 0.03	1.49 ± 0.00	0.39 ± 0.03	-	-	-	-	0.17 ± 0.03	0.50 ± 0.12	-	0.34 ± 0.03
92	1694	1693	germacrone	-	-	-	-	-	-	-	-	-	-	-	-	34.35 ± 5.28	50.73 ± 5.46	56.98 ± 1.13	44.74 ± 3.43	-	-	-	-
93	1763	1759	cyclocolorenone	-	-	-	-	-	-	-	-	-	-	-	-	7.21 ± 2.67	14.02 ± 2.09	6.12 ± 0.55	15.10 ± 1.10	-	-	-	-
94	1971	--	gibbilimbol B	-	-	-	-	-	-	-	-	-	-	-	-	-	-	-	-	7.10 ± 2.74	68.01 ± 7.46	-	9.96 ± 2.53

(Cont. Table S1)

Species	*Piper aduncum*				*Piper amalago*				*Piper caldense*				*Piper mikanianum*				*Piper mollicomum*			
Seasons	**S**	**SM**	**A**	**W**	**S**	**SM**	**A**	**W**	**S**	**SM**	**A**	**W**	**S**	**SM**	**A**	**W**	**S**	**SM**	**A**	**W**
Class of Compounds	**(%)**	**(%)**	**(%)**	**(%)**	**(%)**	**(%)**	**(%)**	**(%)**	**(%)**	**(%)**	**(%)**	**(%)**	**(%)**	**(%)**	**(%)**	**(%)**	**(%)**	**(%)**	**(%)**	**(%)**
Monoterpenes	11.69	16.38	0.82	7.55	40.70	9.82	36.55	32.34	1.02	0.50	5.41	0.22	0.00	0.00	0.00	0.00	32.30	0.28	0.57	5.77
Oxygenated Monoterpenes	4.08	17.32	11.50	11.86	0.00	0.00	0.00	0.00	0.00	0.00	0.00	0.00	0.00	0.00	0.00	0.00	0.00	0.00	0.00	0.00
Sesquiterpenes	14.03	7.32	3.84	6.42	50.80	82.07	49.59	50.70	43.20	18.30	45.90	16.84	17.99	10.79	5.83	8.19	40.76	12.37	24.92	47.62
Oxygenated Sesquiterpenes	8.59	15.78	24.45	14.88	4.82	5.60	5.67	5.63	16.60	5.03	23.16	14.65	41.93	65.12	63.64	60.25	10.54	14.03	50.60	23.28
Arylpropanoids	53.58	32.78	46.31	49.17	0.00	0.00	0.00	0.00	32.46	74.51	16.17	62.30	38.83	22.97	29.87	29.61	1.02	1.32	4.54	4.59
Alkenyl Phenol	0.00	0.00	0.00	0.00	0.00	0.00	0.00	0.00	0.00	0.00	0.00	0.00	0.00	0.00	0.00	0.00	7.10	68.01	0.00	9.96
Total	91.97	89.58	86.92	89.88	96.32	97.49	91.81	88.67	93.28	98.34	90.64	94.01	98.75	98.88	99.34	98.05	91.72	96.01	80.63	91.22

- AI^1 Arithmetic Index calculated; AI^2 Arithmetic Index literature [51]; Relative peak area (%) and Standard deviation of mean (SD); and dashes (—) indicate that the compound was not detected in the sample.

Table S2. Inhibition of *B. cinerea* Growth by Essential Oil from Leaves of *P. aduncum* (Pad), *P. amalago* (Pam), *P. caldense* (Pca), *P. mikanianum* (Pmi), *P. mollicomum* (Pmo), mancozeb (manco), negative control (NC).

		Pad	***Pam***	***Pca***	***Pmi***	***Pmo***	**Manco**	**NC**
	Time	OD	OD	OD	OD	OD	OD	OD
**MIC value**	**1 hour**	0.07 ± 0.01	0.12 ± 0.01	0.06 ± 0.00	0.06 ± 0.00	0.30 ± 0.02	0.11 ± 0.01	0.07 ± 0.00
	**96 hours**	0.08 ± 0.01	0.08 ± 0.00	0.06 ± 0.00	0.06 ± 0.01	0.30 ± 0.01	0.10 ± 0.01	0.54 ± 0.06
**MIC ($\mu L\ mL^{-1}$)**		250	400	20	20	250	40	0

* In the test, three replications were used. OD – optical density. Comparison of Optical Density (OD) in the Antifungal Assay: 1st hour and after 96 hours. Using: Negative Control [Water + Spore Suspension Solution + YPD].
